# A Randomized, Double Blind, Placebo-Controlled, Multicenter Phase II Trial of Allisartan Isoproxil in Essential Hypertensive Population at Low-Medium Risk

**DOI:** 10.1371/journal.pone.0117560

**Published:** 2015-02-18

**Authors:** Ying Li, Xiao-hui Li, Zhi-jun Huang, Guo-ping Yang, Guo-gang Zhang, Shui-ping Zhao, Ying Guo, Shi-juan Lu, Jian-lin Ma, Fan-bo Meng, Ping Chen, Hong Yuan

**Affiliations:** 1 Center of Clinical Pharmacology, the Third Xiangya hospital, Central South University, Changsha, China; 2 Department of Pharmacology, School of Pharmaceutical Sciences, Central South University, Changsha, China; 3 Department of Cardiovascular Medicine, Xiangya Hospital, Central South University, Changsha, China; 4 Department of Cardiology, the Second Xiangya Hospital, Central South University, Changsha, China; 5 Department of Cardiology, Hunan Provincial People’s Hospital, Changsha, China; 6 Department of Cardiology, Haikou City People’s Hospital, Haikou, China; 7 Department of Cardiology, Hainan Provincial People’s Hospital, Haikou, China; 8 Department of Cardiology, China Japan Union Hospital of Jilin University, Changchun, China; 9 Department of Cardiology, Shantou Central Hospital, Shantou, China; Hospital de Clínicas de Porto Alegre, BRAZIL

## Abstract

**Background:**

Angiotensin II receptor blockers (ARBs) is a well-tolerated class of antihypertensive agents, exhibiting effective antihypertensive and cardiovascular protective function. The objective of the study was to examine the efficacy and safety of Allisartan Isoproxil, a newly developed, selective, nonpeptide blocker of the angiotensin II type 1 receptor (AT1R), in essential hypertensive patients at low-medium risk.

**Methods and Findings:**

A Phase II prospective, randomized, double-blind, placebo-controlled, multicenter trial comparing Allisartan Isoproxil 240mg versus placebo was conducted in essential hypertensive patients at low-medium risk at 8 sites in China. After a 2-week placebo baseline period, 275 patients received once-daily treatment with Allisartan Isoproxil 240mg or placebo randomly for 8 weeks. Systolic/diastolic blood pressure (SBP/DBP) was measured at week 2, 4 and 8. By the end of treatment, mean reductions from baseline of SBP and DBP in Allisartan Isoproxil and placebo groups were 14.5/10.4 and 8.3/7.7 mmHg, respectively (P<0.01). The rate of effective blood pressure control in Allisartan Isoproxil group was significantly higher than in placebo group at week 4 (61.3% vs 50.0%, P<0.05) and week 8 (67.2% vs 48.6%, P<0.01). In terms of safety and tolerability, there were no report of death and serious adverse event (SAE) in all subjects. There was no difference of frequency between two groups in adverse event (AE) and adverse drug reaction (ADR) (P>0.05). No one withdraw because of an ADR in two groups. 124 patients received additional 56 weeks treatment with Allisartan Isoproxil and 84 of them completed the study. The rate of effective BP control kept up to 80% since week 24. No significant clinical change was observed and ADRs were generally mild or moderate during the long-term study.

**Conclusions/Significance:**

Allisartan Isoproxil 240mg was effective and safe for essential hypertension patients at low-medium risk.

**Trial Registration:**

http://www.chictr.org/cn/ ChiCTR-TRC-10000886

## Introduction

Hypertension is recognized as a major prevalent risk factor for cardiovascular disease and related death [1]. The prevalence of hypertension was 27.2% in Chinese adult population aged 35 to 74 years [2], while 44.2% in Europe, 27.8% in the US and 27.4% in Canada [3]. It is well known that the renin-angiotensin system (RAS) play a key role in cardiovascular homeostasis including blood pressure (BP) regulation. Angiotensin II, the key effector in RAS, contributes to a range of cardiovascular pathologies and diseases via angiotensin II type-1 receptor (AT1R) activation, while angiotensin II type-2 receptor (AT2R) may mediate protective function [4,5]. Over activation of Angiotensin II in the heart, kidney and vasculature system is one of the most common pathophysiological mechanisms in cardiovascular diseases including hypertension. Angiotensin II receptor blockers (ARBs) represent a relative newer class of antihypertensive agents, developed to exhibit more specific actions and fewer side effects than angiotensin converting enzyme (ACE) inhibitor on original intention [6].

The antihypertensive efficacy of ARBs in patients with mild-to-moderate hypertension has been positively evaluated comparing with ACE inhibitors, beta-blockers, calcium antagonists and diuretics in several studies [7–9]. At the same time, it is demonstrated that ARBs are able to attenuate renal damage associated with hypertension. ARBs also show excellent tolerability evidenced by significant lower incidence of adverse events (AEs) [7, 8, 10].

Losartan potassium was the first non-peptide AT1R antagonist [11]^11^, widely used for hypertension treatment. It can also delay and regress progression of ventricular hypertrophy, heart failure and some kinds of renal disease [12, 13]. Arboxylic acid derivative (EXP3174) is an active metabolite of Losartan potassium which presents its overall activity and has a longer half-life. EXP3174 is a more potent AT1R antagonist with 1000 times affinity binding with AT1R compared with AT2R, resulting in insurmountable antagonism [14]. EXP3174 has been shown to reduce blood pressure after a single intravenous infusion in patients with hypertension [15].

**Fig 1:**
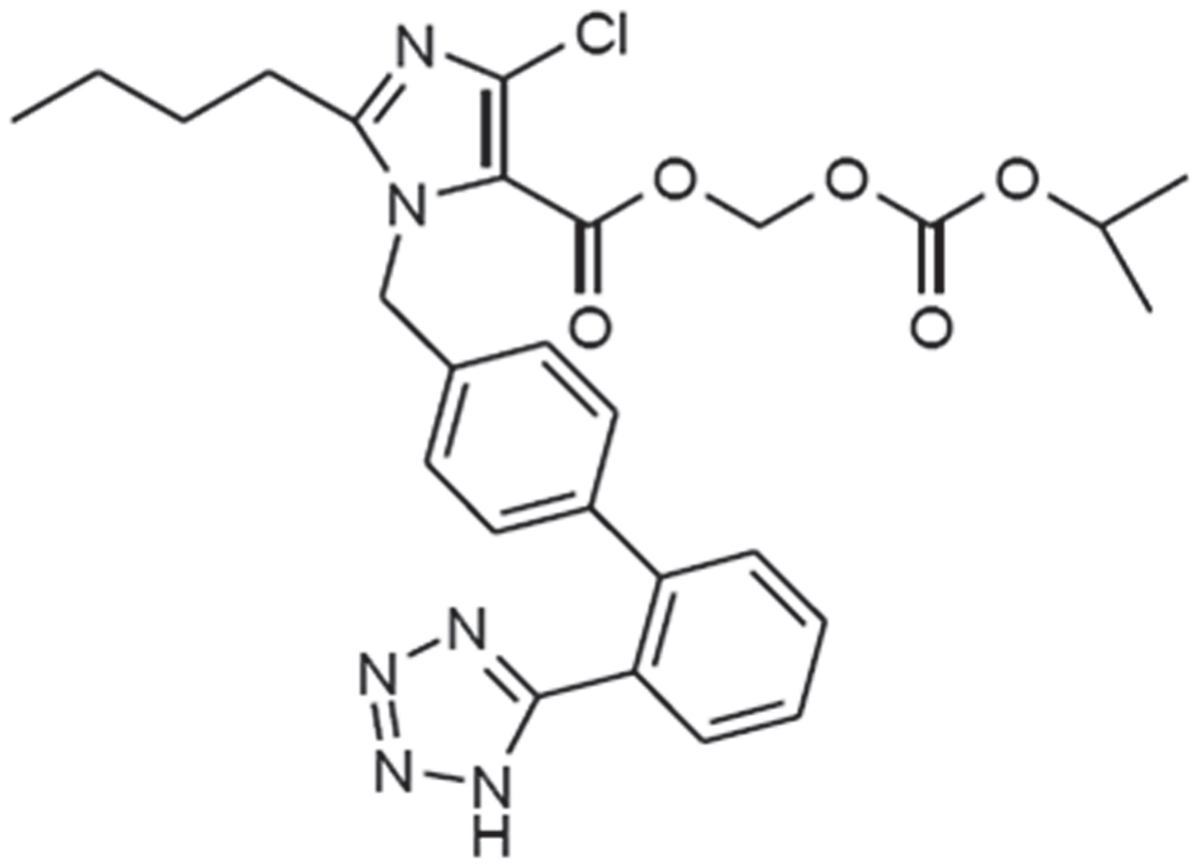
The chemical construction of Allisartan Isoproxil.

Allisartan Isoproxil is developed newly as a prodrug to produce EXP3174 in vivo. Unlike Losartan potassium, EXP3174 is the sole metabolite of Allisartan Isoproxil. After being absorbed in gastrointestinal, Allisartan Isoproxil is hydrolyzed into EXP3174 by esterase completely. Allisartan Isoproxil also has a novel chemical structure which is [(isopropoxycarbonyl)oxy]methyl1-((2′-(1H-tetrazol-5-yl)-[1,1′-biphenyl]-4-yl)methyl)-2-butyl-4-chloro-1H-imidazole-5-carboxylate with the molecular formula of C27H29ClN6O5 and molecular weight of 552.5(Fig. 1). The antihypertensive effect of Allisartan Isoproxil has been conducted in animal models, it is demonstrated that spontaneously hypertensive rats (SHRs) receiving long-term treatment with Allisartan Isoproxil exhibited high efficacy for BP reduction and organ protection with low toxicity [16]. Phase I trial in health volunteers indicated good safety and tolerance of Allisartan Isoproxil at a dose from 20mg to 400mg (data not published).

**Fig 2:**
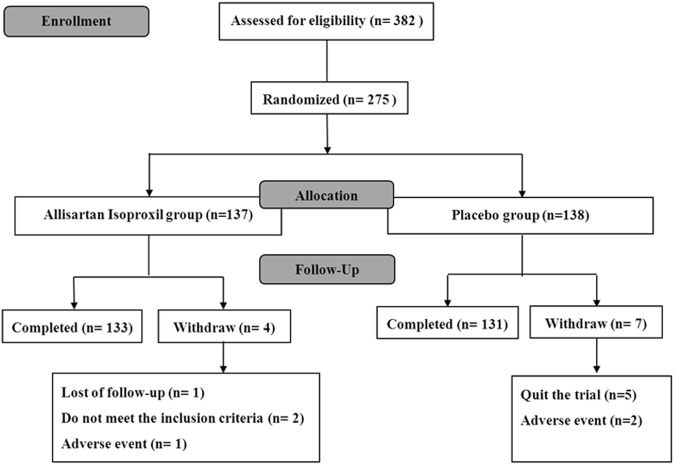
Flow Diagram of Study Patient. The detailed protocol of enrollment, allocation and follow-up research was introduced.

The present 8-week, double-blind, placebo-controlled Phase II trial was designed to characterize the safety and antihypertensive response under once-daily administration of Allisartan Isoproxil 240mg compared with placebo in patients with essential hypertension at low-medium risk. The flow diagram of study was shown in Fig. 2.

## Methods

### Trial design and ethics statement

This study was a prospective, double-blind, Phase II trial with equal parallel cohorts conducted at 8 centers in China to evaluate the safety and efficacy of Allisartan Isoproxil 240mg in essential hypertensive population at low-medium risk. The protocol for this trial and supporting CONSORT checklist are available as supporting information; see S1 CONSORT Checklist and S1 Protocol. This study have been approved by Institutional Review Board (IRB) of the Third Xiangya Hospital, Central South University(No.0915), IRB of Xiangya Hospital, Central South University(No.20090602), IRB of Hunan Provincial People’s Hospital(No.2009–14), IRB of Haikou City People’s Hospital(No.2009–03), IRB of Hainan Provincial People’s Hospital(No.200906), IRB of China Japan Union Hospital of Jilin University(No.2009–06) and IRB of Shantou Central Hospital(No.2009–03) and conducted according to the principles expressed in the Declaration of Helsinki (S1 Table). Written Informed consents have been obtained from the participants. The design of the study, including endpoint, and the statistical method used to define efficacy were all reviewed by the State Food and Drug Administration (SFDA) in China prior to receipt of SFDA authorization for the initiation of the study. The inclusion and exclusion criteria for study enrollment are detailed in S2 Table and meets clinical definitions of Essential Hypertension at low-medium risk according to the 2005 Chinese Guideline on Prevention and Treatment of Hypertension. A list of Principal Investigators in this study is shown in S2 Table along with the 8 study sites and Institutional Review Boards. 7 changes were made on May and Aug 2009. All of those changes had no influence on results (S3 Table). This study recruited first patient on 26th, Jun 2009 and ended follow-up on 22th, Feb 2010 when the last patient completed the study.

Chinese essential hypertensive patients at low-medium cardiovascular risk, aged 18–70 years old, Body Mass Index (BMI) within 18.5–26kg/m^2^, were to be recruited. The study consisted of a 2-week single-blind placebo run-in period followed by an 8-week, double-blind phase. After wash-out period, patients allocated to receive once-daily Allisartan Isoproxil 240mg or placebo. The physical examination and laboratory tests were performed at the baseline and after 8 weeks treatment. Patients attended the clinical investigation ward at week 2, 4 and 8. At each study visit, seated BP and heart rate were measured (Omron), additional laboratory tests (creatinine, electrolyte, aminopherase etc.) were performed at 4 and 8 weeks. Safety evaluation was carried out at each visit. In addition, as long as 56 weeks investigation was carried out of patients’ own free will. Patients taken Allisartan Isoproxil 240mg visited the investigation ward every 8 weeks for safety and efficacy evaluation research. During the long term observation, patients were allowed to take other antihypertensive drugs. The primary efficacy variable for the trial was the changes from baseline in trough mean seated DBP (23 to 26 hours after the morning dose) to the end of the 8-week double-blind treatment period. Secondary end points included as follow: 1) the change of trough mean seated DBP after 4 weeks treatment; 2) the change of trough mean seated SBP after 4, 8weeks treatment; 3) the rate of effective BP control after 4,8 weeks treatment; 4) the change of trough mean seated SBP/DBP after 4, 8weeks treatment in patients with effective response. Effective response was defined as the seated SBP/DBP<140/85mmHg, or seated SBP decrease≥20mmHg and/or seated DBP decrease≥10mmHg.

### Quality assurance of clinical and laboratory data and randomization

Allisartan Isoproxil used in this study was manufactured in accordance with Good Manufacturing Practice (GMP) and tested under Good Laboratory Practice (GLP) and Good Clinical Practice (GCP) guidelines and the ethical principles of the Declaration of Helsinki. Prior to the initiation of the study, vials of active and placebo drug were prepared and labeled with a unique allocation number by the Shenzhen Salubris Pharmaceuticals Co., Ltd. Principal investigators and associate researchers in each study site in charge of enrolling the participants and assigning participants to interventions. After providing informed consent, patients got an allocation number according to the sequence of signing the informed consent by company only. At the site and patient level, only the allocation number was known which provided no insight to the randomization assignment of the study drug. The patient randomization schedules utilized a permuted block design size of 4 and were provided by an independent group. Knowledge of randomization schedules and patient assignments was restricted to the individuals responsible for preparing and packaging the study medication; none of these individuals had any other operational role in the study. Unbinding of the study occurred only on emergency situation defined by center Primary Investigator and final field audits for data accuracy and database lock.

### Sample size

The major end point in research is under superiority design. Set: bilateral alpha = 0.05, beta = 0.20, standard deviation = 7.8 mmHg, expect the difference of antihypertensive effect of two groups is 3 mmHg. Patient distribution proportion is 1:1 in two groups. The minimum sample size is 108 by PASS 2008 software calculation. Considering drop off and other factors, 260 cases in total and 130 in each group should be collected finally. Eight clinical centers participated in this trial with a planned enrollment of 33 patients per site.

### Statistical analysis

All of the analyses were performed using SAS (version 9.13) statistical software. All statistical analyses were two-sided. The data are presented as mean (SD) or median (range). Continuous patient demographic characteristics were compared by ANOVA. The assessment of the primary end point is superior efficacy test between Allisartan Isoproxil and placebo. ANOVA was used for analysis the difference between two groups considering the effect of center of variance. The assessments of the secondary end points were compared by ANOVA, chi-square test and non-parametric test, not considering the effect of center of variance in principle. Safety Assessments was performed between two groups if necessary. Full analysis set (FAS) and Per-Protocol set (PPS) was used for efficacy assessment. According to the principle of intentionality treatment (ITT), all patients who allocated to two groups received therapy and get the efficacy assessment record at least one time were brought into the FAS. PPS included patients who complete the trial and do not violate the protocol. FAS was the major effectiveness evaluation of this study, whereas PPS was for the secondary efficacy assessment. Last observation carried forward (LOCF) was used for estimating the missing value, when FAS under analysis. Key safety assessments included AEs, adverse drug reactions (ADRs), serious adverse events (SAEs) and meaningful change in physical examination, laboratory parameters and electrocardiogram (ECG). ADR was defined as AE that was definitely or possibly related to drugs. Safety Set included the patients who received the therapy at least one time.

## Results

### Patient Characteristics and Accounting

382 patients at low-medium cardiovascular risk were recruited. A total of 275 patients enrolled the double-blind phase of the study. 137 patients were randomized allocated to Allisartan Isoproxil group, while 138 patients to placebo group. By the end of the trial, 258 patients completed the study. Numbers of FAS, PPS and SS and the reasons for discontinuation or protocol deviation were showed in Table 1.

**Table 1:** Numbers of FAS, PPS and SS.

	Allisartan Isoproxil	Placebo	Total
SS	137 (100.0%)	138(100.0%)	275(100.0%)
FAS	137(100.0%)	138(100.0%)	275(100.0%)
PPS	132(96.4%)	126(91.3%)	258(93.8%)
Discontinuation			
Loss to follow-up	1	0	1
Blood pressure not match the inclusive criteria	2	0	2
Quit the Trial	0	5	5
Adverse effect	1	2	3
Protocol deviation			
Take other antihypertensive drugs over 3 times	0	4	4
High fasting blood-glucose, considering diabetes	1	1	2

The patients were generally similar among treatment groups with respect to sex, age, BMI, risk degree of hypertension and baseline SBP and DBP level in both FAS and PPS (Table 2). The study population was predominantly female (57.5%, 158 of 275) and the mean age of patients was 55 years. Mean baseline BP was 151.2/95.4mmHg.

**Table 2:** Patient Demographics and Accounting in FAS.

Entered	Allisartan Isoproxil	Placebo	Total
	N = 137	N = 138	N = 275
Age, mean (SD)	54.2(8.3)	55.4 (9.0)	54.8 (8.6)
Sex, n (%)			
Male	58(42.3)	59(42.8)	117(42.5)
Female	79(57.7)	79(57.3)	158(57.5)
BMI(kg/m^2^), mean (SD)	23.8(2.2)	23.8(2.5)	23.8(2.3)
Risk degree of Hypertension, n (%)			
Mild	34(24.8)	36(26.1)	70(25.5)
Moderate	103(75.2)	102(73.9)	205(74.6)
Baseline blood pressure(mmHg), mean (SD)			
SBP	152.0(10.7)	150.4(9.6)	151.2(10.1)
DBP	95.6(5.0)	95. 2(4.7)	95.4(4.9)

### Primary endpoint analysis

**Fig 3:**
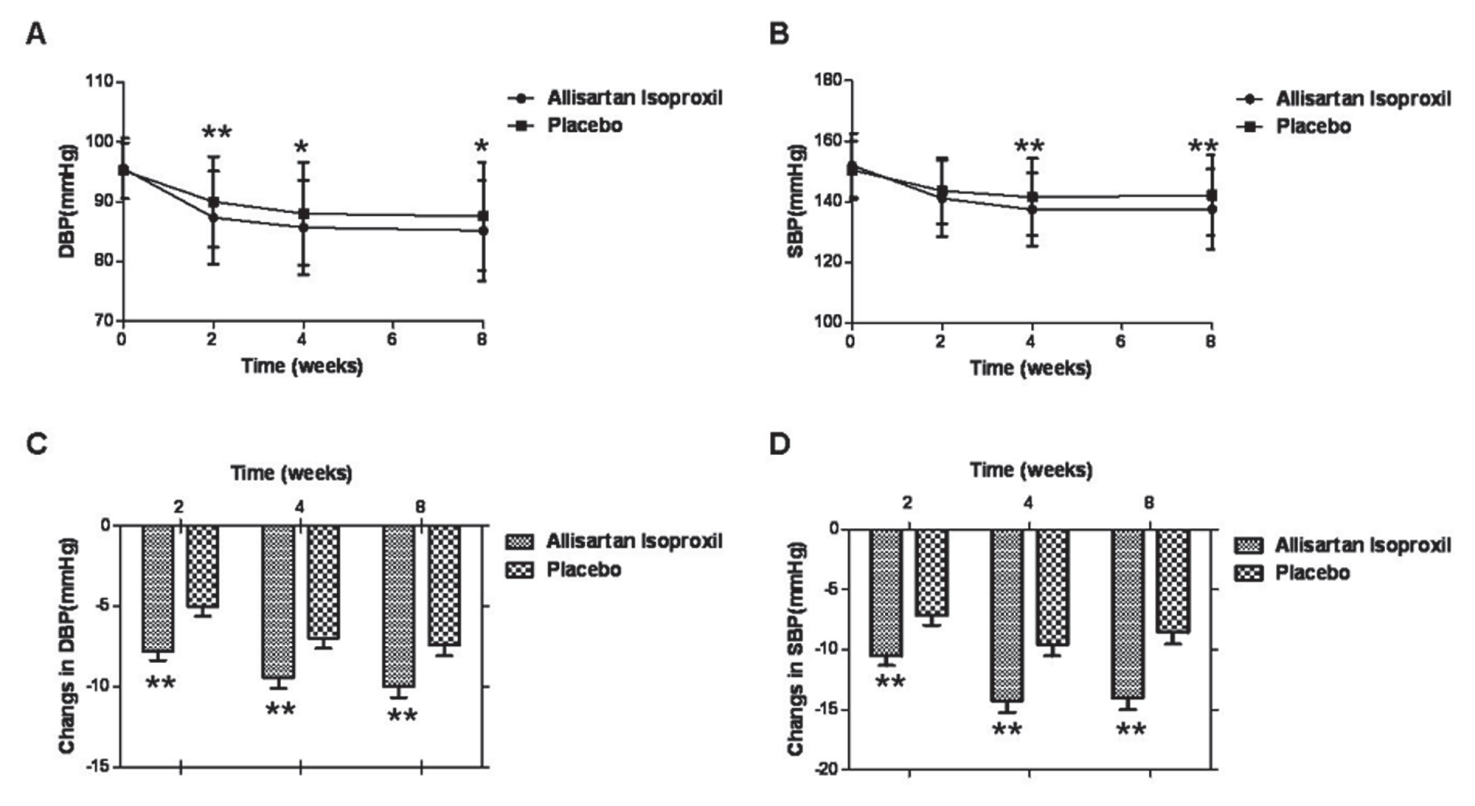
The dynamic changes of clinical DBP and SBP in patient. DBP(A) and SBP(B) at baseline and week 2,4,8 after treatment with Allisartan Isoproxil 240mg and placebo were recorded. The changes of DBP (C) and SBP (D) at week 2,4,8 after treatment with Allisartan Isoproxil 240mg and placebo compareing to baseline were showed. Data are given as mean ±SD.*P<0.05, **P<0.01 compared with the placebo group.

The primary efficacy endpoint of the study was the changes of mean DBP (23 to 26 hours after the morning dose) from baseline to the end of the 8-week treatment. It was 10.4mmHg vs 7.7mmHg (*P*<0.01, 95%CI-4.3, -0.7mmHg) decrease in Allisartan Isoproxil and placebo groups respectively. Changes of DBPs in Allisartan Isoproxil and placebo groups from baseline at week 8 were shown in Fig. 3. Considering the effect of center variance, it was 10.0 mmHg vs 7.4 mmHg (*P*<0.01, 95%CI-4.4, -0.7mmHg) in two groups respectively. Both FAS and PPS showed the same results.

### Secondary endpoint analysis

**Fig 4:**
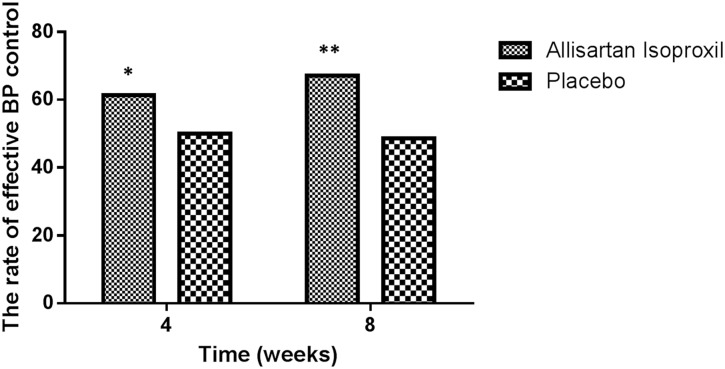
The rate of effective blood pressure control. At week4, 8 after treatment with Allisartan Isoproxil 240mg and placebo, the rate of effective blood pressure control were evaluated, and significant differences existed between two groups. Data are given as mean ±SD.*P<0.05, **P<0.01 compared with the placebo group.

Seated SBPs and DBPs in Allisartan Isoproxil group were significantly lower than placebo group at week 4(137.4 vs 141.5mmHg in SBP, *P*<0.01; 85.7 vs 88.0mmHg in DBP *P*<0.05) and week 8(137.5 vs 142.1mmHg in SBP, *P*<0.01; 85.2 vs 87.6mmHg in DBP *P*<0.05) as shown in Fig. 3. After 4 weeks of treatment, mean reductions of SBP/DBP from baseline in Allisartan Isoproxil 240 mg and placebo subjects were 14.6/9.9 and 8.9/7.2mmHg (*P*<0.01), respectively. After 8 weeks of treatment, mean reductions of SBP from baseline in Allisartan Isoproxil 240 mg and placebo subjects were 14.5 and 8.3mmHg (*P*<0.01), respectively. Changes from baseline of BPs in Allisartan Isoproxil and placebo groups at week 2, 4, 8 were shown in Fig. 3. The rate of effective BP control in Allisartan Isoproxil group was significantly higher than placebo group at week 4 (61.3% vs 50.0% *P*<0.05, 95%CI 0.99–2.62) and 8 (67.2% vs 48.6% *P*<0.01, 95%CI1.36–3.69) as shown in Fig. 4. The change of trough mean seated SBP/DBP after 4 weeks treatment in effective patient were 19.8/13.9mmHg vs 16.1/13.5mmHg (*P*>0.05) in Allisartan Isoproxil and placebo group. After 8 weeks the change of trough mean seated SBP and DBP in effective patient were 18.7 vs 14.9mmHg (*P*<0.05) and 14.2 vs 14.1mmHg in Allisartan Isoproxil and placebo group (*P*>0.05). There were no significant differences of seated DBP and SBP between week 2, 4 and 8 in Allisartan Isoproxil group. Both FAS and PPS showed the same results. No patient withdrew because of ineffective therapy in the Allisartan Isoproxil group.

### Clinical and Laboratory Safety

27.7% patients in Allisartan Isoproxil group were reported at least one AE compared to 25.4% in the placebo cohort (*P*>0.05) (Table 3). The most common clinical AEs occurring during the double blind treatment period (incidence>3% in any one treatment group) were shown in S4 Table. These symptoms were generally mild or moderate. The frequencies of all reported AEs had no difference between Allisartan Isoproxil and placebe group (*P*>0.05). Laboratory measurements indicated that no significant clinical trends were evident for hematology, serum creatinine, potassium, glutamic-pyruvic transaminase (ALT), or glutamic oxalacetic transaminase (AST) test results after 8-week treatment. There were no significant mean changes from baseline for pulse, body weight, or ECG measurements after 8-week treatment among two groups as well. There were no report of death and SAE during the study. ADRs were reported in 8.8% patients in Allisartan Isoproxil groups compared to 10.1% in the placebo cohort in study (*P*>0.05). 11 kinds of ADRs were reported during the double-blind treatment period in Allisartan Isoproxil group, of which the most frequent were hypertriglyceridemia (2.2%), dizziness (2.2%), headache (2.2%), hypercholesteremia (1.5%) and increases in aminopherase (1.5%) (Table 4).

**Table 3:** Summary of adverse events in the study.

	Allisartan Isoproxil	Placebo	All patients	P values
	N = 137	N = 138	N = 275	
Patients ≥ 1 AE	38 (27.7%)	35 (25.4%)	73 (26.5%)	0.68
Patients ≥ 1 ADR	12 (8.8%)	14 (10.1%)	26 (9.5%)	0.84
Patients ≥ 1 SAE	0 (0%)	0 (0%)	0 (0%)	NA
Patients ≥ 1 AE to withdraw the drugs	1 (0.7%)	2 (1.4%)	3 (1.1%)	1.00
Death	0 (0%)	0 (0%)	0 (0%)	NA

**Table 4:** Most Common Clinical adverse drug reactions (incidence >2% in any one group)*.

	Allisartan Isoproxil	Placebo	All patients
	N = 137	N = 138	N = 275
hypertriglyceridemia	3 (2.2%)	2 (1.4%)	5 (1.8%)
dizziness	3 (2.2%)	5 (3.6%)	8 (2.9%)
headache	3 (2.2%)	3 (2.2%)	6 (2.2%)
hypercholesteremia	2 (1.5%)	0 (0.0%)	2 (0.7%)
increases in aminopherase	2 (1.5%)	3 (2.2%)	5 (1.8%)

* The frequency of each adverse drug reactions was comparable in both Allisartan Isoproxil and placebo group (*P*>0.05).

### Long-term safety and efficacy analysis

124 patients in 6 centers entered the additional 56 weeks long term study. The mean age of patients was 54.3±7.6 years and 58.1% of them were female. 124(100.0%), 118(95.2%), 115(92.7%), 115(92.7%), 102(82.3%), 95(76.6%) and 84(67.7%) patients finished 8, 16, 24, 32, 40, 48 and 56 weeks study respectively.

**Fig 5:**
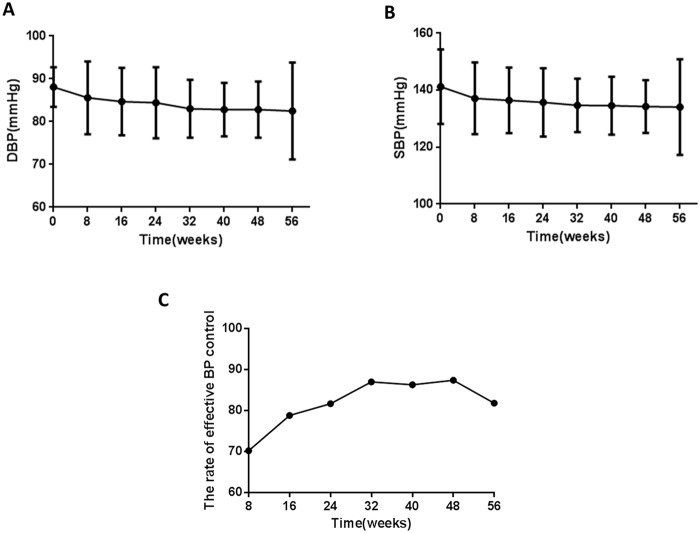
The dynamic changes of clinical DBP/SBP and the rate of effective BP control in the long term observation. DBP(A) and SBP(B) at baseline and week 8, 16, 24, 32, 40, 48 and 56 after treatment with Allisartan Isoproxil 240mg were recorded. The rate of effective BP control (C) were evaluated at week 8, 16, 24, 32, 40, 48 and 56 after treatment with Allisartan Isoproxil 240mg. Data are given as mean±SD.

The baseline BP was 141.2/88.1mmHg after 8 weeks double blind therapy and part of those patients received placebo therapy. 18 patients took other antihypertensive drugs during the long term observation. The dynamic changes of clinical blood pressure and the rate of effective BP control at week 8, 16, 24, 32, 40, 48 and 56 were showed in Fig. 5. Overall, DBP and SBP were significantly decreased after Allisartan Isoproxil therapy and BP reached to a stable state at week 32. The rate of effective BP control was higher than 70% after Allisartan Isoproxil therapy for 8 weeks and kept up to 80% from week 24 to week 56.

There was no report of deaths during the study. Only one SAE (nasopharyngeal carcinoma) reported and considered no drug-related. There were 13 kinds of reported AEs, of which the most frequent were hypertriglyceridemia (4.8%), dizziness (3.2%), hypercholesterolemia (3.2%), increases in aminopherase (3.2%) and increases in low density lipoprotein (3.2%). Laboratory measurements indicated that no significant clinical trends were evident for erythrocyte, leukocyte, platelet, hemoglobin and hematocrit. One patient showed ST-T segment abnormal in ECG test at week 40 but got well at week 56. Only 3 kinds of AEs were recognized as ADRs which including dizziness (1.6%), hypotension (0.8%) and increases in the aminopherase (0.8%). All of the ADRs were generally mild or moderate and self-healing or recovered by treatment.

## Discussion

This is a randomized, double blind, placebo-controlled, multicenter Phase II Trial. Allisartan Isoproxil 240mg induced significant BP reduction (14.5/10.4mmHg) in essential hypertensive patients at low-medium risk and the rate of effective BP control was 67.2% at week 8. Few ADRs was reported in double blind and long term observation period.

Treating hypertension with Angiotensin II receptor blockers (ARBs) was firstly achieved in the 1970s. Compared to other antihypertensive agents targeting RAS such as ACEIs, ARBs induces less cough which is related to bradykinin activation. AT1R selective ARBs has been widely used to lower BP and slow the progression of renal disease, ventricular hypertrophy, heart failure and vascular impairments secondary to hypertension [11–13,17]. Besides classic therapeutic indication of ARBs, other diseases such as chronic graft-versus-host disease may also be a possible indication [18]. EXP3174 is the active metabolite of numerous “sartans”. Developing new ARBs based on EXP3174 is an attractive work.

Allisartan Isoproxil is a new selective, nonpeptide blocker of the AT1R, which is absorbed in gastrointestinal then hydrolyzed into active metabolite EXP3174 by esterase completely. Previous studies in animal models have demonstrated that Allisartan Isoproxil is highly effective for BP reduction [16]. In the present clinical trial, Allisartan Isoproxil also exhibited an effective antihypertensive power. The major finding of this trial was that there were significant reductions in DBP and SBP with Allisartan Isoproxil 240 mg compared with placebo at week 2, 4 and 8 in essential hypertensive patients at low-medium risk. DBP and SBP were reduced since week 2 and showed a highest reduction at week 4 and the antihypertensive effect was maintained as long as 8 weeks. It means that Allisartan Isoproxil can induce a smooth BP reduction.

The rate of effective BP control for Allisartan Isoproxil was 67.2% at week 8 during the randomized, double blind period, which was slightly higher than in other six trials of single antihypertensive drug (hydrochlorothiazide 46%, atenolol 51%, captopril 42%, clonidine 50%, diltiazem 59%, or prazosin 42%) reported before [19]. Another randomized, double blind, phase II clinical study completed in 2009 also revealed that Allisartan Isoproxil showed comparable effect as Losartan potassium (published in chinese). Long term observation indicated that Allisartan Isoproxil even induced a higher rate of effective BP control (>80%) although 18 patients received other antihypertensive drugs. All these results confirmed the effective antihypertensive function of Allisartan Isoproxil. However, only essential hypertensive patients at low-medium risk were recruited in the present study, it was still hard to know the exact effect of Allisartan Isoproxil in hypertensive patients at high cardiovascular disease risk. Clinical trial with combination treatment in such patients would be helpful to answer the question. Anyway, the present study at least provided strong evidences for further clinical research of Allisartan Isoproxil to finally achieve its clinical utilization.

Allisartan Isoproxil appeared to be well-tolerated in this study. There were no serious adverse experiences considered as drug-related, or any withdrew because of an adverse experience in both double blind and long term observation period. There were no apparent differences in the safety profiles observed between subjects treated with Allisartan Isoproxil and placebo. Hyperlipidemia was reported as an ADR in double-blind period but the frequency had no difference between Allisartan Isoproxil and placebo group. And no lipid-metabolism related ADR was reported in long term observation. In fact, a randomized, double blind, phase II clinical study also demonstrated that no lipid-metabolism related ADR was reported in both Losartan potassium and Allisartan Isoproxil group (published in chinese). Previous studies have indicated that BP is correlated with serum cholesterol and triglyceride, suggesting that patients with higher blood pressure values tend to have higher serum cholesterol and triglyceride levels [20–25]. So the dyslipidemia may partly depend on the natural course of hypertension patients and was not drug related. In theory, Allisartan Isoproxil will be hydrolyzed into pharmacological active metabolites EXP3174 by esterase completely without other inactive component which may lead to adverse effect like other “sartans” in the long term treatment. Indeed, previous study showed that the adverse effect frequency in patients received Losartan potassium for 8 weeks was 31.6% including headache, dizziness, nausea and so on [26]. Good safety and tolerance of Allisartan Isoproxil would largely prompt its clinical application.

In addition, it has been proved that ARBs exhibit advantage on hypertension patients with heart failure, diabetic nephropathy, arterial fibrillation and so on [13,27]. But recent studies failed to confirm its benefit on mortality in hypertension patients, especially in patients with diabetes mellitus compared with other antihypertensive medications or placebo [28, 29]. In the present research, all-cause mortality, cardiovascular death and cardiovascular events did not happen during 64 weeks therapy by Allisartan Isoproxil. But it’s still worth to investigate the exact effect on mortality of Allisartan Isoproxil in the future.

A discovery came to notice of this trial was that the maximum BP reduction reached to 8.3/7.7 mmHg in placebo group. First, “regression to the mean” is a statistical phenomenon that subsequent variable will tend to be closer to the average on its baseline. Effective statistical method may provide more accurate estimation of the genuine association between the baseline value of a continuous variable and subsequent change [30]. Second, placebo effect may play an important role in the study. The maximum BP reduction in placebo group was higher than in related previous two studies (2.1/4.0 and 5.3/4.0 mmHg, respectively) [31, 32]. We guess there would be several reasons: 1. The baseline DBP is lower in our study than above-mentioned clinical studies; 2. The positive experience of antihypertensive treatment may influence the placebo effect. 62.3% patients in placebo group have received other antihypertensive drug before the trial, showing higher percentage than above-mentioned clinical studies; 3. The study excluded the complicated hypertension patients who may be insensitive to the treatment including placebo; 4. It is generally accepted that higher temperature may cause vessel dilatation and reduce the blood pressure [33]. While in this study, it is in summer or autumn when most of patients in the placebo group completed the study. Though many people debate placebo treatment and think it is inhumane, it seems necessary to evaluate the true effect of new drugs by comparing with the placebo.

## Conclusion

In conclusion, Allisartan Isoproxil 240 mg produced significant reductions of SBP and DBP in essential hypertension patients at low-medium risk. Meanwhile Allisartan Isoproxil was well-tolerated as evidenced by clinical and laboratory safety profiles. The placebo effect was unexpected high in this study, which suggested the importance that considering placebo effect in clinical trials for new antihypertensive drugs.

## Supporting Information

S1 CONSORT Checklist (DOC)

S1 Protocol (DOC)

S1 Table Clinical Trial Sites, Principal Investigators, and IRB. (DOC)

S2 Table Requirements for Entry into the Phase-II Allisartan Isoproxil Clinical Study. (DOC)

S3 Table List of protocol changes in the study. (DOC)

S4 Table Most Common Clinical Adverse Experiences (incidence >3% in any one group). (DOC)
