# Supplementary material for: A Randomized, Double Blind, Placebo-Controlled, Multicenter Phase II Trial of Allisartan Isoproxil in Essential Hypertensive Population at Low-Medium Risk
Source: PLoS One. 2015 Feb 18;10(2):e0117560. doi: 10.1371/journal.pone.0117560 (PMC4333341; doi:10.1371/journal.pone.0117560)
Supplement: S1 Protocol — (DOC) [file pone.0117560.s002.doc]

**Clinical Trial Protocol**

**A Randomized, Double-Blind, Placebo-Controlled, Multicenter Phase II Clinical Trial of Allisartan Isoproxil in the Clinical Efficacy and Safety Treatment of Primary Hypertention Patients at Low-Medium Risk**

**Protocol number TG0912ALS-3**

**Sponsor:** **Shenzhen Salubris Pharmaceuticals Co.,Ltd**

**Shanghai ALLIST MedicineTechnology Co., Ltd.**

**CRO：Hangzhou Tigermed Co. Ltd.**

**Confidentiality statement**

The proprietary rights of all the information included in the present protocol belong to Shenzhen Salubris Pharmaceuticals Co.,Ltd and Shanghai ALLIST Medicine Science and Technology Co Ltd.. Therefore, it is available only to Researchers, Co-investigator, Ethics Committee and Supervisory and Administration Department and other relevant Medical Institution for review.

Under conditions of not having the written approval of Shenzhen Salubris Pharmaceuticals Co.,Ltd and Shanghai ALLIST Medicine Science and Technology Co Ltd, it is strictly prohibited to let the independent third party into any information except for the signing of the informed consent with the subjects that may participate in the study and giving necessary explanation to them.

**Sponsor**

| Corporate name | Shenzhen Salubris Pharmaceuticals Co.,Ltd  Shanghai ALLIST Medicine Technology Co., Ltd. | | |
| --- | --- | --- | --- |
| Project leader | Zhigang Bi | | |
| Contact address | The 5th floor, No. 1118, Harley Road, Shanghai Zhangjiang high-tech park. | Postal Code | 201203 |

CRO

| Company name | Hangzhou Tigermed Co. Ltd. | | |
| --- | --- | --- | --- |
| Project leader | Qiang Wu | | |
| Contact address | Room 506, No. 6 storied building, Wanda Plaza, No. 93 Jianguo Road, Chaoyang District, Beijing, China | Postal Code | 100022 |

**Data management**

| Company name | Shanghai Tigermed Co. Ltd. | | |
| --- | --- | --- | --- |
| Person in Charge | Fangqiu Xiao | | |
| Contact address | Room 813, Huawen International Building,  Zhongshan West Road No. 999, Shanghai City. | Postal Code | 200051 |

**Statistical analysis unit**

| Company name | Shanghai Tigermed Co. Ltd. | | |
| --- | --- | --- | --- |
| Statistician | Binghua Su | | |
| Contact address | Room 813, Huawen International Building,  Zhongshan West Road No. 999, Shanghai City. | Postal Code | 200051 |

**List of researchers**

| Principal investigator | The Third Xiangya Hospital of Central South University  Professor Hong Yuan | | |
| --- | --- | --- | --- |
| Center Number | 01 | | |
| Address | No. 138, Tongzi Po Road, Yuelu District, Changsha, China. | Postal code | 410013 |

| Co-investigator | Professor Guogang Zhang,  Xiangya Hospital of Central South University | | |
| --- | --- | --- | --- |
| Center Number | 02 | | |
| Address | No. 87, Xiangya Road, Changsha, China | Postal code | 410078 |

| Co-investigator | Professor Shuiping Zhao,  Xiangya No.2 Hospital of Central South University | | |
| --- | --- | --- | --- |
| Center Number | 03 | | |
| Address | No. 139, Middle Renmin road, Changsha City | Postal code | 410011 |

| Co-investigator | Professor Ying Guo,  People's Hospital of Hunan Province | | |
| --- | --- | --- | --- |
| Center Number | 04 | | |
| Address | No. 61, the liberation of West Road, Changsha City, Hunan Province, | Postal code | 410001 |

| Co-investigator | Professor Shijuan Lu,  Peoples Hospital of Haikou | | |
| --- | --- | --- | --- |
| Center Number | 05 | | |
| Address | No. 43, People 's Avenue, Haikou City, Hainan Province | Postal code | 570208 |

| Co-investigator | Professor Jianlin Ma,  People's Hospital of Hainan Province | | |
| --- | --- | --- | --- |
| Center Number | 06 | | |
| Address | No. 19, Xiu Hua Road, Haikou City, Hainan Province. | Postal code | 570311 |

| Co-investigator | Professor Fanbo Meng, China Japan Union Hospital of Jilin University | | |
| --- | --- | --- | --- |
| Center Number | 07 | | |
| Address | No. 2, Sendai Street, Changchun | Postal code | 130033 |

| Co-investigator | Professor Ping Chen, Shantou Central Hospital | | |
| --- | --- | --- | --- |
| Center Number | 08 | | |
| Address | No. 114, Waima Road, Shantou City | Postal code | 515031 |

**Protocol summary**

| **Protocol number** | TG0912ALS-3 |
| --- | --- |
| **Protocol name** | A Randomized, Double-Blind, Placebo-Controlled, Multicenter Phase II Clinical Trial of Allisartan Isoproxil in the Clinical Efficacy and Safety Treatment of Primary Hypertention Patients at Low-Medium Risk |
| **The version number/date** | The 1.2 eidtion, Aug, 06th, 2009 |
| **Sponsor** | Shenzhen Salubris Pharmaceuticals Co.,Ltd  Shanghai ALLIST Medicine Technology Co., Ltd. |
| **Clinical trial staging** | Phase II |
| **Indications** | Medium and lower risks of primary hypertension |
| **Experimental objective** | **The primary objective:**  The primary objective was to compare the change of seated diastolic blood pressure from baseline to 8weeks in primary hypertensive patients with medium and low risks treated with 240 mg Allisartan Isoproxi tablet (ALS-3) and placebo to confirm the absolute antihypertensive effect of the Allisartan Isoproxi tablet (240 mg).  **The secondary objective:**  The secondary objective was to compare the following aspects of primary hypertensive patients at low-medium risk treated with 240 mg Allisartan Isoproxi tablet (ALS-3) and placebo:   - The change of trough mean seated DBP after 4 weeks treatment; - The change of trough mean seated SBP after 4 ,8weeks treatment; - Effective rate after 4,8 weeks treatment (Seated position SBP/DBP＜140mmHg/85mmHg, or SBP decreasing of 20 mm Hg and/or DBP decreasing 10 mm Hg); - The change of trough mean seated SBP/DBP after 4 ,8weeks treatment in effective patient; - Safety evaluation after 8 weeks. |
| Experimental design | A randomized, double-blinded, placebo-controlled, multi center clinical trial |
| **Total number of cases** | There was 1 treatment group and 1 placebo group. One hundred and eight cases of patients in each group can be evaluated, and a total of 216 cases of patients can be evaluated. Considering a loss rate of 20%, a total of 260 subjects were needed to be enrolled. |
| **Research center number** | Eight |
| **Criteria of case selection** | **Inclusion criteria:**   1. Male and female aged 18 to 70 years old; 2. BMI within 18.5-26kg/m^2^; 3. Mild to moderate essential hypertension according to the diagnostic criteria of China (2005); 4. The mean seated blood pressure after 2-week washout period with placebo (the blood pressure should be detected 3 times every 2 minutes after 5-minute resting in sitting position and then take the mean value) should be 140mmHg ≤SBP<180mmHg and 90mmHg ≤DBP< 110mmHg; 5. Able and willing to provide signed informed consent.   **Exclusion criteria:**   1. Having been diagnosed or suspected as secondary hypertension; 2. The difference of systolic blood pressure in seated place measured for three consecutive times is greater than 20 mmHg, and the difference of diastolic pressure is greater than 10 mmHg; 3. Sick sinus syndrome, II~III atrial ventricular block, atrial flutter, atrial fibrillation, or other malignant arrhythmia; 4. Aneurysm in conducting artery or dissecting aneurysm, percutaneous tranluminal coronary angioplasty (PTCA) or other cardiac surgery; 5. Unstable angina pectoris, acute myocardial infarction, cardiac failure, and cerebral accidents within recent 6 months; 6. Asthma or moderate to severe chronic obstructive lung disease; 7. Uncontrolled diabetes, FBG≥11mmol/L or with complications (degenerative nephritis and peripheral neuropathy); 8. Renal arterial stenosis; 9. Renal inadequacy ( Cr>1.5×ULN (the upper limit of the normal range); 10. Serious hepatopathy or hepatic inadequacy (ALT, AST or TBIL> 2×ULN); 11. Electrolyte disturbances (blood potassium or sodium abnormality); 12. Subjects with lower blood volume; 13. Female subjects with pregnancy or in lactation; 14. Subjects with drug or alcohol dependence; 15. Concomitant treatment with other drugs which will affect the blood pressure; 16. Subjects with resistant hypertension; 17. Allergy to any ingredient in the study; 18. attend other clinical trail within 3 month; 19. Suspected as white overcoat hypertension according to the judgment of investigators; 20. Subjects are thought unsuitable for the study by investigators.   **Criteria of subject exiting:**   - - - 1. Withdrawal the study under patients willing will;       2. Occurrence of serious adverse events or intolerable adverse events;       3. Seated SBP ≥ 180 mmHg or seated diastolic blood pressure ≥ 110mmHg during the study;       4. Serious violations of inclusion and exclusion criteria;       5. Poor compliance to the protocal;       6. Lost of follow-up;       7. Other reasons |
| **Research drugs** | The research drug: Allisartan Isoproxi, tablets, 80 mg/tablet;  Placebo: Allisartan Isoproxi placebo, tablets, 80 mg/ tablet. |
| **Grouping of tests** | After 2 weeks washout period (taking placebo), the 260 subjects meeting the inclusion criteria and not meeting the exclusion criteria were randomly assigned to one of the following two groups at a ratio of 1:1:   - The test group A: Allisartan Isoproxi tablet 240 mg (Allisartan Isoproxi tablet 80 mg, 3 tablets), once a day, taking the medicine fasting orally half an hour before breakfast; - The placebo group B: Allisartan Isoproxi tablet placebo 240 mg (Allisartan Isoproxi tablet placebo 80 mg, 3 tablets), once a day, taking the medicine fasting orally half an hour before breakfast;   Patients finished the double blind randomized studies at 8 weeks could participate in additional 56 weeks safety observation under patients own free will.The participants will take Allisartan Isoproxi tablets 240 mg (Allisartan Isoproxi tablets 80 mg, 3 tablets) once a day. |
| Research steps | The present study inlcuded 2 weeks washout period and 8 weeks double-blind randomized treatment period. Interview is needed after treatment for 2 weeks, 4 weeks and 8 weeks. After finishing the double blind randomized treatment period, there is a safety observation period of 56 weeks. There is an interview every 8 weeks.  All the subjects signed the informed consent prior to screening.  Screening/cleaning periods (Interview 1, -3 ~ 0 week)  The starting of giving the treatment with the research drug in the first 3 weeks, and before taking placebo (that is -3 weeks to -2 weeks), the following screening assessment projects should be finished:   - Demographic data including gender and age (date of birth) of the subjects; - Height, weight, waist circumference, smoking and drinking status; - Collecting of the disease history: inquire and record the history of hypertensive disease of the subjects (including the time of illness of hypertension and levels of blood pressure), family history, complications/disease and treatment history, other previous disease and treatment history, allergic history; - Physical examination: conducting of the physical examination of all body systems; - Resting seated blood pressure and measurements of heart rate; - Laboratory and auxiliary examinations: including urine pregnancy test (limited to women of childbearing age), whole blood test, urine routine test test, liver function, kidney function, fasting blood glucose, blood lipid, blood electrolyte and a 12-lead electrocardiogram.   For the patients preliminarily screened as the medium and low risk primary hypertension basically meeting the inclusion criteria but not meeting the exclusion criteria, they entered the placebo washout period of 2 weeks (-2 ~ 0 weeks). The patients will provide and take placebo for 2 weeks. At the same time, diary cards will be given.  In addition, the adverse events and concomitant treatments will be recorded between the signing of the informed consent and taking placebo for 2 weeks.  Baseline (Interview 2, 0 week)  After the subjects took the placebo for 2 weeks, the following aspects should be conducted:   - Measurement of the resting sitting blood pressure and heart rate: the measurement method was as the same as that of the screening period; - Auditing the inclusion/exclusion criteria, the qualified candidates were given random numbers (i.e. drug numbers) according to the order of entering groups from small to large; - According to the random number, the research drugs for the first ~ second week treatment period were provided (including alternative medicine of 3 days); - Recovery of the research drugs of 2 weeks in the placebo washout period; - Transfering and recording of the diary card of subjects; - Recording adverse events and concomitant treatments.   The treatment period (Interview 3, 2 weeks ± 3 days)   - After the subjects taking the research drugs in the double-blind randomized treatment period for 2 weeks, the following aspects were needed to be carried out: - Measurment of the resting sitting blood pressure and heart rate: the measurement method was as the same as that of the screening period; - According to the random number, the research drugs for the third ~ fourth week treatment period were provided (including alternative medicine of 3 days); - Recovery and counting of the remainder research drugs in the first and second week; - Transfering and recording of the diary card of the subjects;   Recording adverse events and concomitant treatments.  The treatment period (Interview 4, 4 weeks ± 3 days)   - After the subjects taking the research drugs in the 4 weeks double-blind randomized treatment period, the following aspects were needed: - Measurement of the resting sitting blood pressure and heart rate: the measurement method was as the same as that of the screening period; - Laboratory examinations: urine routine test, liver function, renal function and serum electrolyte; - The research drugs of the treatment period between the fifth week and the eighth week were distributed according to the random number (including the reserve drug for 6 days); - Recovery and counting of the remainder research drugs in the third and fourth week; - Transfering and recording of the diary card of the subjects; - Recording adverse events and concomitant treatments.   Finishing of the random treatment (Interview 5, 8 weeks ± 3 days)  After the subjects taking the research drugs in the double-blind randomized treatment period for 8 weeks, the following aspects were needed to be carried out:   - Physical examination: physical examination of all body systems was conducted including body weight; - Measurements of the resting sitting blood pressure and heart rate: the measurement method was as the same as that of the screening period; - Laboratory and auxiliary examinations: including urine pregnancy test (only limited to women of childbearing age), whole blood test, urine routine test, liver function, renal function, fasting blood glucose, blood lipid, blood electrolyte and a 12-lead electrocardiogram. - Recovery and counting of the remaining research drugs of the fifth ~ eighth week; - Distribution of Allisartan Isoproxil tablets for 9 weeks’ dose; - Recovery, transfer and recording the diary cards of the subjects; - Recording of the adverse events and concomitant treatments.   Safety observation period (Interview 6-11, 9-56 weeks, one interview for every 8 weeks, the window period ±7 days)  In order to observe the long-term efficacy and safety of the research drugs, all the subjects participated in the research of the safety observation period of 56 weeks on a voluntary basis after they finished the double-blind randomized study of 8 weeks, during which every subject would accept one treatment of 240 mg Allisartan Isoproxi tablet daily. The subjects should visit the research doctor every 8 weeks:   - Measurement of resting sitting blood pressure and heart rate: the measurement method was as the same as that of the screening period; - Laboratory examination was conducted when it was necessary: urine routine test test, liver function, renal function and serum electrolyte; - Allisartan Isoproxil tablets for 9 weeks dose was distributed; - Recoveryand counting of the remainer research drugs of the past 8 weeks; - Transfer and recording of the diary card of subjects; - Recording of the adverse events and concomitant treatments.   In the review 9, namely the review in which the subjects took the research drugs for 32 weeks of the observation period, in addition to the above items, the following aspects were also needed:   - Physical examination: conducting physical examination of all body systems including weight; - Laboratory and auxiliary examinations: including whole blood test, fasting blood glucose, blood lipid, blood electrolyte and a 12-lead electrocardiogram.   After finishing the safety observation period (review 12, the sixty-fourth week ±7 days.)  After finishing the observation period of 56 weeks of treatment for the participants, the following aspects were needed:   - Physical examinations: physical examination of all body systems including body weight; - Measurement of the resting sitting blood pressure and heart rate: the measurement method was as the same as that of the screening period; - Laboratory and auxiliary examinations: including urine pregnancy test (only limited to women of childbearing age), whole blood test, urine routine test, liver function, renal function, fasting blood glucose, blood lipid, blood electrolyte and a 12-lead electrocardiogram. - Recovery and counting of the remaining research drug of the fifty-seventh ~ sixty-fourth week; - Transfer and recording of the diary cards of subjects; - Recording adverse events and concomitant treatment.   Unplanned interview (when the research required or exiting test in advance)  If subjects needed extra interviews or exiting test in advance, the measures that researchers have taken (including the results of laboratory tests) should be recorded on the original records and the unplanned review table in the case report table.  In addition, when the subjects exited tests in advance before the interview 5 or after they entered the safety observation period and ended early safety observation period, the following aspects should be conducted as far as possible:   - Physical examination: physical examination of all body systems; - Measurement of the resting sitting blood pressure and heart rate: the measurement method was as the same as that of the screening period; - Laboratory and auxiliary examination: including urine pregnancy test (only limited to women of childbearing age), whole blood test, urine routine test test, liver function, renal function, fasting blood glucose, blood lipid, blood electrolyte and a 12-lead electrocardiogram; - Recovery and counting of all the remaining research drugs; - Recovery, transfer and recording of the diary cards of subjects (only applicable to the subjects during the double-blind research period); - Recording adverse events and concomitant treatment. |
| **Therapeutic effect index** | **Primary outcome parameters:**   - The changes from baseline in trough mean seated DBP (23 to 26 hours after the morning dose) to the end of the 8-week double-blind treatment period   **Secondary outcome parameters:**   - The change of trough mean seated DBP after 4 weeks treatment - The change of trough mean seated SBP after 4 ,8weeks treatment - Effective rate after 4,8 weeks treatment - The change of trough mean seated SBP/DBP after 4, 8weeks treatment in effective patient - Note: Effective respond was defined as the seated SBP/DBP<140/85mmHg, or seated SBP decrease≥20mmHg and/or seated DBP≥10mmHg. |
| **Safety index** | Adverse events included the abnormal demonstrations of the clinical symptoms after medication and the changes of clinical significances of physical examination, laboratory examination and ECG. |
| **Statistical analysis** | **Estimation of sample sizes:**  This is a superior efficacy test study. The efficiency test was conducted on the main therapeutic effect index. Setting bilateral α = 0.05, *β* = 0.20, and the standard deviation SD = 7.8 mmHg, the difference of the antihypertensive effect in the two groups were detected as *D* = 3 mmHg. The distribution ratio of the patient cases in the two groups was 1:1. The estimated minimum sample size was 108 pairs. Taking into account of the factors such as dropping, the present research plan included 260 cases into the two groups with 130 cases of patients in the experiment group and the control group, respectively.  **Statistical analysis of the data set:**  Statistical analysis was conducted by using the full analysis set, per-protocol population set and safety data set. The quality control was strictly carried out in the whole process of the data statistics to ensure the authenticity of the assessment of data.  Full analysis set (Full analysis set, FAS): according to the fundamental principle of intention to treat (ITT), all the randomly enrolled subjects with more than one medication administration record and effective evaluation were included in the full analysis set. When selecting full analysis set for analysis, the latest observation LOCF (LOCF) estimation method was adopted for the estimation of the missing values. Full analysis set is the main effectiveness method for the evaluation of population in the present study.  The Per-Protocol set (PPS): PPS included all the subjects with completed prescribed treatment protocal or no serious violations of tests. The exact definition of the serious violations of the scheme will ultimately determined in the data auditing, which generally included the following situations (but they are not limited to these cases): Not meeting the inclusion criteria, having interference treatment after being selected, poor compliance, interview beyond the time window. PP set is a secondary analysis of the crowds of the effectiveness, but if the results were inconsistent with the full analysis set, detailed analysis should be done on the inconsistent results.  Safety data set (Safety Set, SS): the definition of safety data set was the subjects that at least received one time drug treatment.  **Statistical analysis method:**  Statistical analysis was conducted by using the SAS9.13 statistical analysis software for calculation. Quantitative description of the parameters included the calculation of mean, standard deviation, median, minimum value and maximum value. The description of the classified index employed the case number and percentage of the cases.  Descriptive statistics were conducted on the completion of the study. The comparison among groups was conducted on the demographic and baseline characteristics to analyze the equilibrium between groups.  Analysis of the main outcome measures: the main efficacy index will use the test of superiority. In addition, the dereasee value os DBP of each subject after treatment of 8 weeks will also be calculated, and descriptions in each group were done (mean, standard deviation, median, minimum, maximum). Comparisons between groups used analysis of variance considering center effect.  Analysis of the secondary outcome indicators: the center effect was not considered in principle. Comparisons between groups employed the variance analysis, chi-square test or nonparametric statistical method.  Analysis of the safety evaluation: parameters of safety evaluation included adverse events/ adverse drug reaction (ADR)/important adverse events or serious adverse events, physical examination and laboratory examination and a 12-lead electrocardiogram. Detailed description was conducted on the cases of adverse events during the test. The incidence rates of the adverse events between the two groups were summarized and compared. The indexes of laboratory examination were statistically described. The difference between two groups was compared when it was necessary. A detailed description of the laboratory indexes and normal cases before treatments was conducted.  This study did not conduct interim analysis. |

**Experimental flow chart**

|  | **Screening and cleaning periods** | | | **Baseline period** | **The treatment period (window period ± 3 days)** | | | **Safety observation period ^5^ (window period ± 7 days)** | | | |
| --- | --- | --- | --- | --- | --- | --- | --- | --- | --- | --- | --- |
|  | **Screenig** | | **Cleaning** |  |  |  |  |  |  |  |  |
| Interview times | **1** | | | **2** | **3** | **4** | **5** | **6-8** | **9** | **10-11** | **12** |
| Interview time (week) | **－3～－2** | **－2～0** | | **0** | **2** | **4** | **8** | **9-32** | **40** | **48-56** | **64** |
| Signing written informed consent | **X** |  | |  |  |  |  |  |  |  |  |
| Collecting Medical History | **X** |  | |  |  |  |  |  |  |  |  |
| Physical examination | **X** |  | |  |  |  | **X** | **X^6^** | **X** | **X^6^** | **X** |
| Blood pressure and heart rate in sitting position | **X** |  | | **X** | **X** | **X** | **X** | **X** | **X** | **X** | **X** |
| Auditing inclusion/exclusion criteria | **X** |  | | **X** |  |  |  |  |  |  |  |
| Urine pregnancy test ^1^ | **X** |  | |  |  |  | **X** |  |  |  | **X** |
| Routine blood test ^2^ | **X** |  | |  |  |  | **X** | **X^6^** | **X** | **X^6^** | **X** |
| Routine urine test ^3^ | **X** |  | |  |  | **X** | **X** | **X^6^** | **X** | **X^6^** | **X** |
| Liver function（ALT、AST、  TBil） | **X** |  | |  |  | **X** | **X** | **X^6^** | **X** | **X^6^** | **X** |
| Renal function（Bun、Cr、  Ua） | **X** |  | |  |  | **X** | **X** | **X^6^** | **X** | **X^6^** | **X** |
| Blood fat（TG、TC、HDL、LDL） | **X** |  | |  |  |  | **X** | **X^6^** | **X** | **X^6^** | **X** |
| Fasting blood-glucose（FPG） | **X** |  | |  |  |  | **X** | **X^6^** | **X** | **X^6^** | **X** |
| Blood electrolytes（K、Na、  Cl） | **X** |  | |  |  | **X** | **X** | **X^6^** | **X** | **X^6^** | **X** |
| 12 electrocardiograph（ECG） | **X** |  | |  |  |  | **X** |  | **X** |  | **X** |
| The drug release of the cleaning period ^4^ | **X** |  | |  |  |  |  |  |  |  |  |
| The drug recovery of the cleaning period |  |  | | **X**^4^ |  |  |  |  |  |  |  |
| 收Release and recovery of the research drugs |  |  | | **X** | **X** | **X** | **X** |  |  |  |  |
| Drug release and recovery of the safety observation period |  |  | |  |  |  | **X** | **X** | **X** | **X** | **X** |
| Release, recovery and transcription of diary cards of the subjects | **X** | **X** | | **X** | **X** | **X** | **X** |  |  |  |  |
| Recording concomitant medication | **X** | **X** | | **X** | **X** | **X** | **X** | **X** | **X** | **X** | **X** |
| Recording adverse events | **X** | **X** | | **X** | **X** | **X** | **X** | **X** | **X** | **X** | **X** |

1. It applies only to women of childbearing age.

2. Whole blood test indexes include the following aspects: total numbers of erythrocytes and leukocytes, and percentages of neutrophile granulocytes and lymphocytes, platelet count, hemoglobin and hematocrit.

3. The indexes of routine urine detection inlcude the following aspects: urine leukocytes, urine erythrocytes and urine proteins and carbohydrate.

4. The released drugs of the cleaning period can meet applciation for 17 days. The cleaning period was 14 days. The participants must be back to the hospital in 14 ± 3 days for baseline interview.

5. Interview was conducted once every 8 weeks, and a total of 7 interviews were carried out.

6. If there is abnormity of the laboratory examinations in the previous double-blind period, reexamination must be conducted. If it is normal in the previous results, the researchers can decide whether to inspect according to their own circumstances;

Note: according to the conditions of the patients, the researchers can increase the safety inspection of the subjects at any time.

**Table of contents**

[1 Research backgrounds](#__RefHeading___Toc228092694) 22

[1.1 Introduction of diseases](#__RefHeading___Toc228092695) 22

[1.2 introduction to drugs](#__RefHeading___Toc228092696) 23

[2 research OBJECTIVE](#__RefHeading___Toc228092697) 36

[2.1 primary objective](#__RefHeading___Toc228092698) 36

[2.2 secondary objective](#__RefHeading___Toc228092699) 36

[3 the expected duration of research, the number of Research Center and the case number of Planning](#__RefHeading___Toc228092700) 37

[4 entry and exit of criteria](#__RefHeading___Toc228092701) 37

[4.1 inclusion criteria](#__RefHeading___Toc228092702) 37

[4.2 exclusion criteria](#__RefHeading___Toc228092703) 37

[4.3 exit criteria](#__RefHeading___Toc228092704) 38

[5 Research design](#__RefHeading___Toc228092705) 39

[5.1 Research types and design principles](#__RefHeading___Toc228092706) 40

[5.2 Randomization and blinding](#__RefHeading___Toc228092707) 40

[5.2.1 Randomized methods](#__RefHeading___Toc228092708) 40

[5.2.2 Rules of breaking blinding of the randomized double-blind trial](#__RefHeading___Toc228092709) 40

[5.3 Experimental steps and stages](#__RefHeading___Toc228092710) 41

[5.3.1 Screening/Cleaning (Interview 1, -3 ~0 weeks)](#__RefHeading___Toc228092711) 41

[5.3.2 Baseline period (Interview 2, 0 week)](#__RefHeading___Toc228092712) 42

[5.3.3 The treatment period (Interview 3, 2 weeks ± 3 days)](#__RefHeading___Toc228092713) 42

[5.3.4 The treatment period (Interview 4, 4 weeks ± 3 days)](#__RefHeading___Toc228092714) 43

[5.3.5 Finishing of random therapy (Interview 5, 8 weeks ± 3 days)](#__RefHeading___Toc228092715) 43

[5.3.6 Safety observation period (Interview 6-11, 9-56 weeks, 1 interview every 8 weeks, window period ±7 days)](#__RefHeading___Toc228092716) 44

[5.3.7 Finishing of the safety observational period (Interview 12, the 64th week±7 days)](#__RefHeading___Toc228092717) 45

[5.3.8 The unplanned outside interview (research needs or early exit of test in advance)](#__RefHeading___Toc228092718) 45

[6 Research drugs](#__RefHeading___Toc228092719) 46

[6.1 Drug name, dosage form](#__RefHeading___Toc228092720) 46

[6.2 Medication methods](#__RefHeading___Toc228092721) 46

[6.3 storage condition](#__RefHeading___Toc228092722) 46

[6.4 Drug packaging and labeling](#__RefHeading___Toc228092723) 46

[6.4.1 Packaging of drugs in wah-out period](#__RefHeading___Toc228092724) 46

[6.4.2 Packaging of drugs druing the treating period](#__RefHeading___Toc228092725) 46

[6.4.3 labels of the research drugs](#__RefHeading___Toc228092726) 47

[6.5 Drug distribution and checking](#__RefHeading___Toc228092727) 47

[6.6 The principle of dose selection and dose adjustment](#__RefHeading___Toc228092728) 48

[7 Treatments of subjects](#__RefHeading___Toc228092729) 49

[7.1 Research drugs](#__RefHeading___Toc228092730) 49

[7.2 Combined application of drugs](#__RefHeading___Toc228092731) 49

[7.3 Subject compliance](#__RefHeading___Toc228092732) 50

[8 efficacy assessments](#__RefHeading___Toc228092733) 50

[8.1 Primary outcome](#__RefHeading___Toc228092734) 50

[8.2 Secondary outcome](#__RefHeading___Toc228092735) 51

[9 safety assessment](#__RefHeading___Toc228092736) 51

[9.1 safety index](#__RefHeading___Toc228092737) 51

[9.1.1 physical examination](#__RefHeading___Toc228092738) 51

[9.1.2 Laboratory and auxiliary examination](#__RefHeading___Toc228092739) 51

[9.2 adverse events](#__RefHeading___Toc228092740) 52

[9.2.1 Definition of adverse events](#__RefHeading___Toc228092741) 52

[9.2.2 Observation, recording and reporting of adverse events/serious adverse events](#__RefHeading___Toc228092742) 52

[9.2.3 Criteria of severity degrees of adverse events/seriou adverse events](#__RefHeading___Toc228092743) 52

[9.2.4 Criteria of relationship between experimental drugs of adverse events/seriou adverse events...................................](#__RefHeading___Toc228092744) 52

[9.2.5 The treatment, follow-up and duration of cases of adverse events/important adverse events 53](#__RefHeading___Toc228092745)

[9.2.6 The judgment and treatment parameter abnormalities of laboratory tests](#__RefHeading___Toc228092746) 53

[9.3 Serious adverse event](#__RefHeading___Toc228092747) 54

[9.3.1 Definition of serious adverse events](#__RefHeading___Toc228092748) 54

[9.3.2 Recording and report procedure of serious adverse events](#__RefHeading___Toc228092749) 54

[9.3.3 Treatment, mode of follow-up and duration of the occurrence of serious adverse events](#__RefHeading___Toc228092750) 54

[9.3.4 Measures of subjects after pregnancy](#__RefHeading___Toc228092751) 55

[10 Data management](#__RefHeading___Toc228092752) 55

[10.1 The traceability of data, and fill in and hand over of case report form (CRF)](#__RefHeading___Toc228092753) 55

[10.2 Design and establishment of database](#__RefHeading___Toc228092754) 55

[10.3 data encode](#__RefHeading___Toc228092755) 55

[10.4 Input and modification of data](#__RefHeading___Toc228092756) 56

[10.5 data verification](#__RefHeading___Toc228092757) 56

[10.6 lock and Unblinding of the database](#__RefHeading___Toc228092758) 56

[11 statistics](#__RefHeading___Toc228092759) 56

[11.1 sample size estimate](#__RefHeading___Toc228092760) 56

[11.2 definition and selection of analytic set](#__RefHeading___Toc228092761) 57

[11.3 hypothesis test](#__RefHeading___Toc228092762) 57

[11.4 statistical method](#__RefHeading___Toc228092763) 58

[11.4.1 Analysis method](#__RefHeading___Toc228092764) 58

[11.4.2 Interim analysis](#__RefHeading___Toc228092765) 59

[12 Experimental management](#__RefHeading___Toc228092766) 59

[12.1 declaration](#__RefHeading___Toc228092767) 59

[12.2 Ethical part](#__RefHeading___Toc228092768) 59

[12.3 The verification of original data](#__RefHeading___Toc228092769) 59

[12.4 Quality control and assurance](#__RefHeading___Toc228092770) 60

[12.5 Informed consent/data protection protocol](#__RefHeading___Toc228092771) 60

[12.6 modification of Clinical program](#__RefHeading___Toc228092772) 60

[12.7 Case report form](#__RefHeading___Toc228092773) 60

[12.8 Clinical Research Monitor](#__RefHeading___Toc228092774) 61

[12.9 privacy of subjects](#__RefHeading___Toc228092775) 61

[12.10 publications](#__RefHeading___Toc228092776) 61

[12.11 archiving of data](#__RefHeading___Toc228092777) 61

[13 appendix](#__RefHeading___Toc228092778) 62

[13.1 appendix 1： Standard of diagnosis and risk stratification of blood pressure (Guidelines for the prevention and treatment of hypertension in China 2005 Edition)](#__RefHeading___Toc228092779) 62

[13.2 Measurement methods of blood pressure and heart rate at resting sitting position in clinic](#__RefHeading___Toc228092780) ....................................................................................................................................................67

[13.3 label of research drugs](#__RefHeading___Toc228092781) 69

[14 references](#__RefHeading___Toc228092782) 73

[15 Signature page of protocol](#__RefHeading___Toc228092783) 74

**Abbreviation table**

The followings are the phrases and jargons used in the present research protocol:

| Abbreviation and terminology | Explanation |
| --- | --- |
| AE | Adverse event |
| ALT | Alanine transaminase |
| AST | Aspartate aminotransferase |
| Bun | Blood urea nitrogen |
| Cr | Creatinine |
| CRF | Case report form |
| CRO | Contract research organization |
| DBP | Diastolic pressure |
| ECG | Electrocardiogram |
| FAS | Full analysis set |
| FPG | Fasting plasma glucose |
| GCP | Good Clinical Practice |
| HDL | High density lipoprotein |
| ICH | International Conference on Harmonization of |
| IEC | Independent ethics committee |
| ITT | Intent-to-treat |
| LDL | Low density lipoprotein |
| LLN | Lower limits of normal |
| PPS | Per-protocol population |
| SAE | Serious adverse event |
| SBP | Systolic blood pressure |
| SOP | Standard operating procedure |
| SS | Safety data set |
| TBil | Total bilirubin |
| TC | Total cholol |
| TG | Triglyceride |
| Ua | Uric acid |
| ULN | Upper limit of normal |

# Research background

## Introduction of the disease

Hypertension is one of the commonest cardiovascular diseases in the world, which often causes complications of heart, brain, kidney and other organs and seriously harms the health of human beings. The prevalence rate of hypertension in China is increased significantly. The data of the “National nutrition and health survey of residents in China” organized by the Ministry of health in 2002 demonstrated that the number of the patients with hypertension has reached 160000000 and China has become one of the countries with the most serious harm of hypertension in the world.

The main purpose of the treatment of hypertension is to minimize the total risk of the cardiovascular morbidity and mortality^[1-3]^. Application of the effective antihypertensive drugs to reduce the elevated arteriotony of the patients with hypertension can significantly decrease the morbidity and mortality caused by hypertension. The meta-analysis of randomized clinical trials and the related blood pressure-reducing test with the vascular events or deaths as the destination provides a series of evidence for the treatment of hypertension. Compared with that of the control group with placebo, the treatment of the hypertension patients in the systole and the diastole with antihypertensive drug can reduce 42% of the the relative risk of the brain stroke, 14% of the the relative risk of the coronary events and 14% of the the relative risk of the total mortality. The treatment of the hypertension patients in the single systole with antihypertensive drug can reduce 30%, 23% and 13% of the above events, respectively ^[1]^.

**Strategy for the treatment of hypertension**

The stratification was conducted according to the low risk, medium risk, high risk or very high risk:

After the examination of the patients and the comprehensive assessment of the total risk profile, the patients were determined whether they belonged to low risk, medium risk, high risk or very high risk.

For the patients with high risk and very high risk: no matter how the economic conditions they were in, medication must be immediately conducted for the hypertension, the coexisted risk factors and the clinical conditions;

For the patients with medium risk, the blood pressure and other risk factors of the patients must be observed for several weeks to further understand the situations of the patients, and then it was decided whether to carry out medication.

For the patients with low risk: the patients were observed for quite some time, and then it was decided whether to start the medication.

At present, the antihypertensive drugs for the treatment of hypertension mainly include the following five categories, namely diuretics, β-blockers, angiotensin converting enzyme inhibitors (ACEI), angiotensin receptor blockers (ARB), calcium antagonists, which all can be used as the initial drug use and maintenance therapy of the antihypertensive therapy. Based on the importance of the renin-angiotensin system (RAS) in the hypertension and etiology and pathology of the cardiovascular diseases, the development of RAS inhibitors from every side become one of the main directions of the researches of cardiovascular drugs currently and the next period of time. The clinically available drugs acting on RAS mainly include two types presently: ACEI and ARB. Recently, FDA newly approved a renin inhibitor of Novartis ®, so the drugs acting on RAS also was added a new type.

The antihypertensive effect of ARB is reliable. In addition to the role of lowering blood pressure, ARB also has other benefits as shown in the below:①Reduce hypertrophy of left ventricle; ②improve function of diastole; ③reduce ventricular arrhythmia; ④improve endothelial dysfunction; ⑤improve compliance of large artery;⑥reduce microalbuminuria; ⑦ protect or improve renal function except for the patients with severe congestive heart failure or renal artery stenosis; ⑧improve insulin sensitivity of insulin; ⑨reduce level of serum uric acid (only in losartan). The prominent characteristics of the ARB are that the side effect is little without first dose hypotension but with occasional dizziness, headache, fatigue, diarrhea, abdominal pain, joint pain, and it does not increase with the increasing of dose. Compared with that of ACEI, calcium antagonists and β-blockers, the patients have better tolerance with ARB and can be used in long term.

Because of its prominent advantages in safety and effectiveness, ARB has become a hot spot for the research and development of the antihypertensive drugs. Since the first available oral administration of ARB losartan has been approved for clinical application in 1994, then there are a number of "sartans" class of drugs that have been developed to accommodate, Such as valsartan (valsartan), Maher Bbe Chatain (irbesartan), eprosartan, candesartan, tasosartan, telmisartan, olmesartan, of which because of its side effects, tasosartan has been withdrawn^[4-11]^. Therefore, developing a new antihypertensive drug of ARB with safe and reliable antihypertensive effect will provide a better treatment option for the majority of patients with hypertension.

## Introduction of drugs

***Introduction of the research of drugs***

Allisartan Isoproxil (Allisartan Isoproxil, ALS-3) is the 1.1 kind of oral antihypertensive drug of chemical medicine independently researched and developed by Shenzhen Salubris Pharmaceuticals Co.,Ltd  and Shanghai ALLIST Medicine Science and Technology Limited Company, belonging to the antagonist of the angiotensin II receptor (AT_1_ receptor).

Allisartan Isoproxil was completely hydrolyzed by enzyme into the only pharmacologically active metabolites of EXP3174 in the absorption process by gastrointestinal tract *in vivo* gastrointestinal absorption. Allisartan Isoproxil can selectively interact with the receptor of AT1 and can block all the physiological functions related to AngⅡregardless of the source of or synthesis pathway of AngⅡ. However, Allisartan Isoproxil does not play roles independent of the block of AT_1_ receptors, such as the enhancement of bradykinin mediated-effects (cough, bronchial spasm, angioneurotic edema and other common ACE inhibitors-related adverse effects are associated with bradykinin savings).

***Pre clinical study***

The comprehensive pre clinical studies of Allisartan Isoproxil (pharmaceutical research and pharmacological and toxicological study) showed that the security of Allisartan Isoproxil was high, and the anti-hypertensive effect was good. Compared with that of the losartan potassium, Allisartan Isoproxil has significant advantages, and its quality is controllable.

**（1）** The non-clinical and pharmacodynamics characteristics: The anti hypertensive effect of Allisartan Isoproxil was good, and its application in rat, canine and many model animals all have been obtained and confirmed very well.

The experimental results of the spontaneously hypertensive rats (SHR) with acute (single drug administration) pressure-reduced test, long term (consecutive 4 months) treatment test and the acute antihypertensive test of the vascular hypertension rats of 2 kidney 1 clip renal showed that: Rats treated with intragastric administration of Allisartan Isoproxil in the dose ranges of 7.5-30mg/kg demonstrated dose-dependent antihypertensive effects, but the heart rate was not affected at the same time of the decreasing of blood pressure. Furthermore, after long-term administration of Allisartan Isoproxil, Allisartan Isoproxil reversed SHR left ventricular hypertrophy, eased renal (cortical) atrophy and prevented aortic thickening. The acute hypotensive test of the Beagle dogs with hypertension induced by Angiotensin II demonstrated that: Single intragastric administration of Allisartan Isoproxil in the dose ranges of 5-20mg/kg showed good dose-dependent antihypertensive effects, but the heart rate was not affected at the same time of the decreasing of blood pressure. Hemodynamic experiments of dogs indicated that Allisartan Isoproxil showed no significant effect on the left ventricular systolic pressure, left ventricular end diastolic pressure (LVEDP), left ventricular peak systolic and diastolic change rate of the anesthetic dog.

**（2）non clinical pharmacokinetic characteristics:**

After intragastric administration with Allisartan Isoproxil in rats, the main metabolite by hydrolysis was EXP3174, and the prototype drug was not detected in the blood (determination by LC/MS/MS method, and the lower limit of quantification of 1ng/mL). The dynamic process of EXP3174 in rats and beagle dogs conformed to the two-compartment model. C_max_ and AUC_0-t_ value and dose exhibited relationship of direct proportion in the dose ranges between 10-90 mg/kg it rats. In the dog dose range of 3-27 mg/kg in dogs, the values of C_max_ and AUC_0-t_ were lower than that of the direct proportion with the increase of dose. The absolute bioavailability of the Active Pharmaceutical Ingredient (API) ALS-3 in rats and dogs were 4.11% and 7.38%, respectively. The absolute bioavailability of the tablet in dogs was 15.5%, higher than that of the API. There was no accumulation of drugs in bodies of rats and dogs after multiple dosing. The metabolite EXP3174 is widely distributed in tissues, and the distributed tissues mainly include liver, intestinum tenue and intestinum crassum, stomach, spleen and lung. The content of EXP3174 in brain tissue is very low, suggesting that the drug is not easy to pass through blood brain barrier. EXP3174 has very high binding rate of plasma protein, and its average binding rate of plasma protein of rats is 100%, and its average binding rate of plasma protein of human is 99.6%. The research results of metabolisms showed that the olysis of Allisartan Isoproxil occurs rapidly in bodies of rats and EXP3174 is produced. The major metabolic pathways of EXP3174 are N-oxidation and hydroxylation. After intragastric administration with ALS-3 in rats, EXP3174 and the prototype form are mainly excreted from feces.

**（3）Evaluation studies of pre clinical safety:**

Allisartan Isoproxil with concentrations of 40-400 mg/kg demonstrated no significant effect on the general behavior and spontaneous activity of mice. Allisartan Isoproxil with concentrations of 4.5-135 mg/kg illustrated no obvious effect on blood pressure, heart rate, cardiac rhythm, frequency and amplitude of respiratory in dogs. The maximum tolerance doses of the single intragastric administration of Allisartan Isoproxil in mice and dogs were >10 g/kg and > 3g/kg, respectively. The toxicity test of 26 weeks long term of SD rats displayed that 20 mg/kg/day was the non-toxic response dose in rats, and 80 mg/kg/day was the toxic response dose in rats. Kidney was the toxicity target organ of the long term toxicity test of SD rats. The toxicity mainly exhibited that the parameters of erythrocytes were decreased slightly, the blood urea nitrogen of male rat was increased, the absolute and relative weights of heart were decreased, and the basophilia of epithelial cells of renal tubular was degenerated (common and spontaneous lesions). The long-term toxicity test results of dogs with withdrawal of drugs for 6 weeks in the 39^th^ week showed that the dose of ≤500 mg/kg/day was the safe dose, which was approximately 33 times that of the effective dose of 15mg/kg in rats and 50 times that of the effective dose of 10 mg/kg in dogs. The toxicity mainly exhibited that the color of feces showed offwhite after drug administration, and the parameters of blood were slightly reduced. Allisartan Isoproxil had no mutagenic effect: the results of bacterial reverse mutation test (Ames test), chromosome aberration test of CHL cells and micronucleus tests of bone marrow in mice were all negative. The toxicity test of the sensitive period of teratogenesis of the SD rats treated with intragastric administration of Allisartan Isoproxil indicated that the non-toxic effect dose of intragastric administration of ALS-3 on parent population and embryonic development of the SD pregnant rats with gestation of D6-D15 was 90 mg / (kg•d), which was 6 times that of the pharmacodynamics dose of rats, while 270mg/ (kg•d) was he slightly toxic dose. ALS-3 has no carcinogenicity and dependence.

***Phase I clinical study***

Phase I clinical study was carried out on the healthy Chinese volunteers by Phase I Clinical research laboratory, Clinical Pharmacology Center, Peking Union Medical College Hospital.

**Tolerance research of administration of single dose:** The single center, randomized, double-blind, placebo-controlled design was employed in the present investigation. There were 6 groups with successively increased doses of Allisartan Isoproxil tablets of 20 mg, 50 mg, 90 mg, 150 mg, 240 mg and 400 mg. Starting from the lowest dose group, each dose group was studied successively. There was at least an interval of 7 days between two groups. Four enrolled subjects took medicine first in the group of 20 mg. After observation and evaluation, experiments were carried out on the subjects continuously in the following dosage groups. Eight subjects were randomly assigned in the rest every dose group, 6 of whom took Allisartan Isoproxil tablets, and another 2 people took the corresponding placebo. The results demonstrated that it was safe for the healthy subjects received a single oral Allisartan Isoproxil tablet (20 ~ 400mg) is safe without occurrence of adverse events associated with kidney. Adverse events might be related to the research drugs were the grade 1 leukopenia (1 case, 150 mg dosage group), grade 2 bradycardia (1 case, 250 mg dosage group). These adverse events could be healed themselves without treatment, showing a transient characteristic.

**Pharmacokinetic study of administration of single dose:** Pharmacokinetic study contained 2 tests: the comparison test with losartan potassium and single dose pharmacokinetics test. The comparison test with losartan potassium employed randomized, open, single dose, two-period crossover trial design. The healthy volunteers were randomly divided into two groups: the single dose crossover oral equimolar of 100 mg losartan potassium or 120 mg Allisartan Isoproxil tablets. Single dose pharmacokinetics test of Allisartan Isoproxil tablets used the randomized, open, three-period crossover Latin square experimental design. The healthy volunteers were randomly divided into 6 groups, who received single oral dose of Allisartan Isoproxil tablets of 60 mg, 120 mg and 240 mg according to the different orders. The HPLC MS/MS was used in determination of the concentration of human plasma drug in the above two experiments, and the data of pharmacokinetic parameters were obtained by the winNonLin software. Experimental results: 1) Pharmacokinetics test comparing with losartan potassium: after the subjects orally took Allisartan Isoproxil tablets with dose of 120mg, the active compound of Aili the Candesartan cilexetil was not detected in blood (a detection sensitivity of 0.5 ng/ml). The concentration of EXP-3174 in blood reached peak Tmax median of 2.5 (1-3.5) in about 2 hours. T_1/2_ was 8.94 ± 2.26 h, Cmax was 484 ± 289 ng/mL, AUC_0-last_ was 2.72 ± 0.99 hr*mg/L, AUC_0-inf_ was 2.73 ± 0.99 hr*mg/L; After orally taking Losartan Potassium with a dose of 100 mg for the subjects, the concentration of EXP-3174 in blood also reached peak Tmax median of 2 (1.33-4) in about 2 hours. T_1/2_ was 8.81 ± 3.08 h, Cmax was 1050 ± 308 ng/mL, AUC_0-last_ was 4.76 ± 1.01 hr*mg/L, and AUC_0-inf_ was 4.78 ± 1.01 hr*mg/L. After taking Losartan Potassium, the concentration of Losartan Potassium in blood reached peak Tmax median of 2 (1.33-4) in about 1 hour. T_1/2_ was 2.27 ± 0.31 h, Cmax was 641 ± 330 ng/mL, AUC_0-last_ was 0.729 ± 0.2 hr*mg/L, and AUC_0-inf_ was 0.736 ± 0.2 hr*mg/L. 2) Single dose pharmacokinetics test of three doses of Allisartan Isoproxil : the corresponding three doses were 60 mg, 120 mg and 240mg. After taking single oral dose of Allisartan Isoproxil tablet, the pharmacokinetic parameters of EXP-3174 in blood were T_max_ median: 1.84（1-3）h, 2.01（1.33-3）h and 2.5（1.67-3）h, Cmax: 207±76.4 ng/mL, 493±284ng/mL and 737±201 ng/mL，T_1/2_: 9.63±2.82h, 10±3.39h and 9.94±2.45h, AUC_last_: 1.33±0.34 hr*mg/L, 2.62±1.01 hr*mg/L and 4.43±0.83 hr*mg/L, AUC_0-inf_: 1.35±0.34 hr*mg/L, 2.63±1.01 hr*mg/L and 4.45±0.83 hr*mg/L. **Test conclusion:** The pharmacokinetic studies of the comparisons with losartan potassium showed that Allisartan Isoproxil and losartan potassium had similar EXP-3174 peak reaching time (Tmax about 2 hours) and half-life (T_1/2_ of about 10 hours); Under conditions of equimolar drug administration, the exposure value EXP-3174 of Allisartan Isoproxil (AUClast and Cmax) was about half of corresponding value of losartan potassium. The single dose pharmacokinetic studies of Allisartan Isoproxil demonstrated that the pharmacokinetic parameters of EXP-317 AUC_last_ and Cmax increased with the increasing of dose of the drug (60-240 mg) after Allisartan Isoproxil administration, which was basically linear. The dosage effect on Tmax and T1/2 was not obvious. In addition, these two studies illustrated that the tolerance of the healthy subjects to Allisartan Isoproxil was good in the dose range of 60~240mg.

**Test of the effect of food:** Twelve healthy subjects were randomly selected. The study was divided into 2 stages. According to cross, randomized and open methods, Allisartan Isoproxil tablets of 120 mg were given to the subjects in the fasting and postprandial states. In 30 min, 1, 1.5, 2, 2.5, 3, 4, 6, 8, 12, 24, 36 and 48 h before and after administration of the drug, blood samples were collected to detect the plasma concentration of EXP-3174. Research results displayed that the average AUC_last_ and C_max_ of EXP-3174 of the healthy subjects with single oral administration of Aili the Candesartan cilexetil tablets of 120 mg after meal were about 64.5% and 61.6% of that of the single drug taking of fasting, indicating that the bioavailability of the orally administration of Allisartan Isoproxil tablets after meal was lower than that of the fasting state medication. It is safe for the healthy subjects receiving a single oral Aili medoxomil tablet (120mg), and no adverse event was observed.

**Research on the tolerance and pharmacokinetics of multiple dose drug adminstrations:** According to the random, double blind, placebo controlled method, 12 subjects were randomly enrolled (the proportion of either gender was not less than 1/3) with daily administration of 240mg Allisartan Isoproxil tablets or placebo once (randomly assigned 10 subjects with oral administration of 240 mg Allisartan Isoproxil tablets, the other 2 subjects orally took the corresponding placebo) for 7 consecutive days. Blood samples were collected at each time point before administration of the drug, in 48 hours after the first day and ninth day of drug administration, and the time points between the fourth days to the eighth day for the analysis of plasma concentration of EXP-3174. Adverse events were assessed throughout the study period. Research results showed that the healthy subjects were safe daily for single oral administration a dose of Allisartan Isoproxil tablets (240mg) for 7 consecutive days, and there was no grade 2 or above adverse event was observed (There was a total of 2 cases of grade 1 adverse event that might be realted to drug: 1 case of increased ALT and 1 case of vertigo); Under the conditions of healthy subjects daily single dose administration of 240 mg Allisartan Isoproxil tablet for 7 consecutive days, there was no accumulation of drug in the body and the self induction or inhibition effects of the drug enzyme.

***Clinical research***

**General situation of the test**

The present protocol was bid by Shanghai Ellis Medicine Technology Co., Ltd.. The approval number of the Allisartan Isoproxil (ALS-3) by the State Drug Administration for the treatment of patients with mild to moderate primary hypertension was 2007L05207. The randomized, double-blind, double-dummy, multi-dose, positive drug parallel control and multicenter phase II clinical study for testing of clinical efficacy and safety was carried out in ten national research centers from August of 2008 to January of 2009.

**Materials and methods**

**The research subjects**

1. Inclusion criteria: (1) Ages of 18-70 years old, gender not limited. (2) 18.5kg/m^2^ ≤ BMI ≤ 30kg/m^2^（BMI=body weight/length^2^）. （3）After 2 weeks placebo washout period, the average sitting blood pressure (after sitting and resting for 5 minutes, three measurements of systolic/diastolic blood pressure every 2 minutes, and the average value was obtained) was 140mmHg ≤ SBP < 180 mmHg and 90 mmHg ≤ DBP < 110mmHg.（4）Signing of the written informed consent.

2 Exclusion criteria: (1) known or suspected to be of secondary hypertension. (2) the sick sinus syndrome, II~III degree atrioventricular heart-block, atrial flutter and atrial fibrillation and other malignant or potentially malignant arrhythmia. (3) aneurysm or aortic dissection, percutaneous transluminal coronary angioplasty or heart surgery. (4) unstable angina, acute myocardial infarction, heart failure, cerebral vascular accident history within the past 6 months. (5) the blood glucose is not controlled very well. fasting blood glucose greater than or equal to 11 mmol/L, or with complications (nephropathy, peripheral neuropathy). (6) known renal artery stenosis. (7) renal, hepatic insufficiency. (8) electrolyte disorder (blood potassium, sodium disorders). (9) needing to take other medicines that affecting blood pressure in addition to the research drug during the test.

3. Exiting criteria: (1) exiting of subjects voluntarily. (2) Occurrence of seious adverse events or unacceptable adverse events. (3) Seriously breach of inclusion and exclusion criteria. (4) Poor compliance to the protocal. (5) Lost of interview.

**Research drugs**

Allisartan Isoproxil tablet (80mg), Allisartan Isoproxil placebo tablet (80mg) and losartan potassium placebo (50mg) were produced by Shenzhen Salubris Pharmaceuticals Co.,Ltd and the Shanghai ALLIST pharmaceuticals Co.Ltd manufactures. Losartan potassium tablets (50mg) were produced by Hangzhou MSD Pharmaceutical Co., ltd.. The experiment used double-blind and double simulation technology.

**Research methods**

The present study was a randomized, double-blind, multi dose, positive drug-parallel control and multi-center clinical experiment.

Research Center: the test was carried out in 10 research centers, and the principal investigator was Professor Ningling Sun from the Peking University People's Hospital. The other 9 research centers were Chinese Academy of Sciences respectively Hospital of Peking Union Medical College Hospital, China-Japan Friendship Hospital, General Hospital of PLA, 301 Hospital, Shanghai Jiao Tong University Affiliated Sixth People's Hospital, the second hospital of Tianjin City, the Third Xiangya Hospital of Central South University Medical College, Qilu Hospital of Shandong University, Tongji Hospital Affiliated to Huazhong University of Science and Technology and the second hospital affiliated to Zhejiang University.

2. Research steps: (1) screening period: physical examination, history review, electrocardiographic examination and laboratory examination were conducted on the patients. All patients discontinued the original antihypertensive drugs and the forbidden drugs of the present study, and took Allisartan Isoproxil and losartan potassium placebo once daily for 2 weeks. Whether the subjects met the inclusion and exclusion criteria was assessed again at the end of the test. (2) the treatment period: the qualified subjects were randomly assigned to the groups of Allisartan Isoproxil of 80mg, Allisartan Isoproxil of 160mg, Allisartan Isoproxil of 240mg and 50 mg potassium losartan treatment group at a ratio of 1:1:1:1 once a day, 4 pills every time for taking 8 weeks. Laboratory examination was conducted at the fourth week in the treatment period. ECG and laboratory examination were carried out at the eighth week in the treatment period. Compliance of application of the drugs, all adverse events and drug combination were recorded.

3. Evaluation parameters: (1) the main outcome parameters: changes in the relative baseline value of the sitting blood pressure after 8 weeks. (2) the secondary outcome parameters: changes in the relative baseline value of the sitting blood pressure after 4 weeks; the percentage of the subjects meeting the standard of the sitting blood pressure after 4 and 8 weeks; 4. changes in the relative baseline value of the sitting blood pressure of the subjects meeting the standard of the sitting blood pressure after 8 weeks. Reaching the standard of the sitting blood pressure is that SBP/DBP < 140mmHg/85mmHg, or decreasing of SBP by 20mmHg and/or decreasing of DBP by 10mmHg. (3) safety indicators: adverse events/ADR/important adverse events/serious adverse events including the abmormal demonstrations of the clinical symptoms after medication and the changes with clinical significances such as signs of life, physical examination, laboratory examination and ECG.

4. Statistical analysis: the full analysis set, per-protocol population set and safety set were used. The description of the quantitative parameters would calculate mean, standard deviation, median, minimum value and maximum value. The description of the classification parameters used case numbers and percentage of cases. General statistical test used two-tailed test, and one-tailed test needed explaining. A P value less than or equal to 0.05 was considered as whether there was statistical significance criteria for judging differences.

Analysis of the main outcome parameters: the decreasing values of SBP/DBP in each subject treated for 8 weeks would be calculated. Various parameters including mean, standard deviation, median, the minimum value, and the maximum value would be described by grouping. The comparisons between groups were analyzed by variance analysis considering center effects.

Analysis of the secondary outcome parameters: the center effect was not considered in principle. The variance analysis, chi square test or nonparametric statistical method were used in the comparison between groups.

Analysis of safety evaluation: safety parameters included adverse events/ADR/important adverse events or serious adverse events, physical examination and laboratory examination and a 12-lead electrocardiogram. Occurrence of cases of adverse events during the test process was described in detail. The incidence rate of the occurrence of adverse events between the two groups were summarized and compared. The indexes of laboratory examination were statistically described, and the varied differences between the two groups were compared when it was necessary. A detailed description of the laboratory indexes before treatment and abnormal cases of treatment was conducted.

**Results**

**Effective results:**

This study selected a total of 401 cases of patients with mild to moderate primary hypertension. After cleaning for 2 weeks, 283 subjects were randomly enrolled into groups, of whom there were 34 cases of dropping (12%) 7 cases were excluded (2.5%). The full analysis set included 279 cases (Allisartan Isoproxil with doses of 80, 160, 240mg and losartan of 50mg included 69, 68, 72, and 70 cases). The PPS included 246 cases (Allisartan Isoproxil with doses of 80, 160, 240mg and losartan of 50mg included were 60, 58, 67, 61 cases).

In FAS analysis, the sitting blood pressure (systolic/diastolic blood pressure) of the subjects treated with Allisartan Isoproxil of 80, 160, 240mg and losartan of 50mg for 4 weeks was 11.33/6.96, 11.35/6.35, 10.85/8.39, mmHg and 11.41/7.67mmHg less than that of the relative baseline. The sitting blood pressure (systolic/diastolic blood pressure) in various groups treated for 8 weeks was 9.12/6.30, 11.04/7.22, 11.76/8.57mmHg and 12.39/8.41mmHg was less than that of the relative baseline. The absolute decreasing value of the blood pressure increased with the increasing dose of Allisartan Isoproxil . The absolute decreasing value of the Allisartan Isoproxil (240 mg) was similar to that of the Losartan (50 mg), but there were no significant difference between groups.

After the treatment with Allisartan Isoproxil 80, 160, 240 mg and losartan of 50mg for 4 weeks, the percentrate of the standard meeting of the sitting blood pressure were 57.97%, 42.65%, 59.72% and 52.86%. The average decreasing values of the sitting blood pressure were decreased by 14.15/9.40, 15.17/11.41, 14.07/11.74mmHg and 18.57/12.84mmHg comparing with that of the baseline, but there was no significant difference between groups. After 8 weeks of treatment, compliance percentages of the sitting blood pressure were 43.48%, 52.94%, 58.33% and 54.29%, which were dropped by an average of 18.63/12.33, 20.06/13.22, 17.69/13.02 mmHg and 19.39/14.11mmHg comparing with that of the baseline, but there were no significant differences between groups.

90.32% of the subjects were mild hypertension. After treatment for 8 weeks with Allisartan Isoproxil with concentrations of 80, 160, 240mg and losartan of 50mg, the average sitting blood pressures were decreased by 8.18/5.84, 11.14/7.71, 12.48/8.82 and 12.22/7.89 mmHg comparing with that of the baseline.

After 8 weeks of treatment, the normal recovery percentages of the sitting blood pressure (SBP/DBP < 140mmHg/90mmHg) in the groups of Allisartan Isoproxil with concentrations of 80, 160, 240mg and losartan of 50mg were 39.13%, 47.06%, 50% and 44.29%. The average decreasing value of the sitting blood pressures were 18.07/12.11, 20.78/13.25, 18.03 /11.75mmHg and 20.42/13.65mmHg comparing with that of the baseline, but there were no significant differences among groups.

PP analysis was consistent with that of the FAS analysis results.

**Safety results:**

This study used a total of 282 cases of subjects with at least one drug treatment, who were all included in the safety analysis set, of whom the groups of Allisartan Isoproxil with 80 160, 240mg and losartan of 50mg were 70, 69, 73, 70 cases, respectively.

**（1）Adverse events**

The incidence rates of adverse events in the groups of Allisartan Isoproxil with 80 160, 240mg and losartan of 50mg were 32.86% (23/70), 27.54% (19/69), 30.149% (22/73) and 35.71% (25/70), mainly manifested as dizziness, headache, nausea, dizziness, abnormal blood lipid (including cholol, low density lipoprotein, glycerol three ), dysfunction of liver, urine protein and urine leucocyte abnormalities, of which the incidence rate of dizziness was the highest. In addition, the Allisartan Isoproxil (240mg) group had no abnormal blood lipids. All adverse events mainly demonstrated mild, some were moderate, which could be alleviated themselves or alleviated after symptomatic treatments. There was no significant difference in the incidence rate of adverse events and severe degrees among groups.

**（2）Adverse reactions**

The incidence rates of the adverse reactions in the groups of Allisartan Isoproxil with 80 160, 240mg and losartan of 50mg were 8.57% (6/70), 4.35% (3/69), 9.59% (7/73) and 10% (7/70), mainly manifested as headache, dizziness, headache, nausea, abnormal liver function, of which the incidence rates of headache (4.11%) and headache (2.74%) in the group of the Allisartan Isoproxil (240mg) were the highest. The incidence rates of dizziness, abnormal liver function abd nausea in each dose group were similar. The adverse reactions mainly exhibited as mild, some were moderate, which could be alleviated themselves or alleviated after symptomatic treatments. There was no significant difference in the incidence rate of adverse reactions and severe degrees among groups.

**（3）Important adverse events**

The incidence rates of the important adverse events in the groups of Allisartan Isoproxil (80, 160, 240mg) and losartan group (50mg) were 11.43% (8/70), 14.49% (10/69), 10.96% (8/73) and 15.71% (11/70), mainly manifested as headache, dizziness, headache, nausea, abnormal liver function. There was no significant difference in the incidence rate of he important adverse events among groups.

**（4）Serious adverse events**

In this study, a total of 1 case of serious adverse event was discovered, and the incidence rate of the serious adverse event was 1.43%. There was no significant difference between the experimental groups (P=0.7411). The case of patient has sudden cholecystalgia (the patient with previous gallstone disease history). The patient was in hospital and cholecystectomy was conducted on him. The researchers determined that the serious adverse event might not related to the research drug, so the drug was discontinued.

**（5）Laboratory tests, vital signs and physical examination**

After 8 weeks of treatment, the Allisartan Isoproxil group (160mg) had decreased neutrophil percentage, and increased percentrage of lymphocyte (statistically significant), but there was no significant difference among the groups in comparisons. There were significant differences in the platelet count between groups before and after treatments. After 8 weeks of treatment, the platelet count in the group of Losartan (50mg) increased and there was a significant difference. There was no statistical difference in the group of various doses of Allisartan Isoproxil before and after treatments.

After 8 weeks of treatment, triglyceride in the groups of Allisartan Isoproxil with 160 mg and 240 mg and the losartan group with 50 mg all decreased. The total cholol in the Allisartan Isoproxil group (80mg) was elevated. The high density lipoprotein in the Allisartan Isoproxil (240mg) group and the losartan group (50mg) were both increased, and there were significant differences. There was no significant difference of the above indexes before and after treatment between groups. In addition, there was no case of normal blood glucose, lipid and total cholol before treatment and abnormal after treatment on the above parameters only in the Allisartan Isoproxil group (240mg), but other experimental groups al had the case.

There was no significant difference in various indexes of liver functions before and after treatment and between groups, and there was no case with dysfunction of liver after treatment in the group of Allisartan Isoproxil (240 mg). After treatment with Allisartan Isoproxil (240 mg) for 4 weeks and 8 weeks, the level of creatinine was decreased (statistically significant); After treatment for 4 weeks, levels of uric acid in the groups of various doses of Allisartan Isoproxil and the group of Losartan (50 mg) were all reduced (statistically significant). After treatment for 8 weeks, levels of uric acid in the groups of Allisartan Isoproxil (80 mg, 240 mg) and the group of Losartan (50 mg) were all decreased (statistically significant); but there was no significant difference between the groups. After treatment for 4 weeks, levels of serum sodium and chlorine both increased in the group Losartan (50 mg), which was significantly different from that of the groups with different doses of Allisartan Isoproxil when comparing, but there was no significant difference between these groups in 8 weeks; the concentration of potassium was elevated after treatment with Allisartan Isoproxil (80 mg, 240 mg) for 4 weeks (statistically significant). The level of serum potassium was increased after treatment only with Allisartan Isoproxil (80 mg) for 8 weeks, but there was no significant difference between groups.

Of all the subjects with normal electrocardiogram before treatment, there were 2 patients had abnormal electrocardiogram treated for 8 weeks, and the related adverse events were recorded, which was diagnosed by 1 researcher that it was not related to the research drug, while another research believed that it was related to the research drug. There was no significant difference in the resting heart rate between different groups after treatments for 2, 4, 8 weeks.

# Research objective

## Primary objective

The primary objective was to compare the change of seated diastolic blood pressure from baseline to 8weeks in primary hypertensive patients with medium and low risks treated with 240 mg Allisartan Isoproxi tablet (ALS-3) and placebo to confirm the absolute antihypertensive effect of the Allisartan Isoproxi tablet (240 mg). Therapeutic effect index is the changes from baseline in trough mean seated DBP (23 to 26 hours after the morning dose) to the end of the 8-week double-blind treatment period

## Secondary objective

The secondary objective was to compare the following aspects of primary hypertensive patients at low-medium risk treated with 240 mg Allisartan Isoproxi tablet (ALS-3) and placebo:

- The change of trough mean seated DBP after 4 weeks treatment;
- The change of trough mean seated SBP after 4 ,8weeks treatment;
- Effective rate after 4,8 weeks treatment (Seated position SBP/DBP＜140mmHg/85mmHg, or SBP decreasing of 20 mm Hg and/or DBP decreasing 10 mm Hg);
- The change of trough mean seated SBP/DBP after 4 ,8weeks treatment in effective patient;
- Safety evaluation after 8 weeks.

# The expected duration of research, the number of research center and of the case number of planning

The present research program is planned to enroll subjects from May of 2009, and finish grouping in August of 2009, and statistical analysis and summary will be conducted in December of 2009.

Research will be carried out in 8 National Research center. The study designs 1 treatment group and 1 placebo group. One hundred and eight cases of patients in each group can be evaluated, so a total of 216 cases of patients are needed to be evaluated. Considering a shedding rate of 20%, a total of 260 qualified cases of subjects with hypertension are needed to be enrolled.

# Entry and exit of criteria

## Inclusion criteria

All the subjects who meet all of the following conditions are enrolled in this experiment:

Inclusion criteria:

1. Male and female aged 18 to 70 years old;

2. BMI within 18.5-26kg/m2;

3. Mild to moderate essential hypertension according to the diagnostic criteria of China (2005);

4. The mean seated blood pressure after 2-week washout period with placebo (the blood pressure should be detected 3 times every 2 minutes after 5-minute resting in sitting position and then take the mean value) should be 140mmHg ≤SBP<180mmHg and 90mmHg ≤DBP< 110mmHg;

5. Able and willing to provide signed informed consent.

## Exclusion criteria

The subjects meeting one of the following conditions will not be selected in the test:

1. Having been diagnosed or suspected as secondary hypertension;

2. The difference of systolic blood pressure in seated place measured for three consecutive times is greater than 20 mmHg, and the difference of diastolic pressure is greater than 10 mmHg;

3. Sick sinus syndrome, II~III atrial ventricular block, atrial flutter, atrial fibrillation, or other malignant arrhythmia;

4. Aneurysm in conducting artery or dissecting aneurysm, percutaneous tranluminal coronary angioplasty (PTCA) or other cardiac surgery;

5. Unstable angina pectoris, acute myocardial infarction, cardiac failure, and cerebral accidents within recent 6 months;

6. Asthma or moderate to severe chronic obstructive lung disease;

7. Uncontrolled diabetes, FBG≥11mmol/L or with complications (degenerative nephritis and peripheral neuropathy);

8. Renal arterial stenosis;

9. Renal inadequacy ( Cr>1.5×ULN (the upper limit of the normal range);

10. Serious hepatopathy or hepatic inadequacy (ALT, AST or TBIL> 2×ULN);

11. Electrolyte disturbances (blood potassium or sodium abnormality);

12. Subjects with lower blood volume;

13. Female subjects with pregnancy or in lactation;

14. Subjects with drug or alcohol dependence;

15. Concomitant treatment with other drugs which will affect the blood pressure;

16. Subjects with resistant hypertension;

17. Allergy to any ingredient in the study;

18. attend other clinical trail within 3 month;

19. Suspected as white overcoat hypertension according to the judgment of investigators;

20. Subjects are thought unsuitable for the study by investigators.

## Exiting criteria

The subjects can withdraw from the experiment at any time. The exiting reasons are mainly as follows:

1. Withdrawal the study under patients willing will;

2. Occurrence of serious adverse events or intolerable adverse events;

3. Seated SBP ≥ 180 mmHg or seated diastolic blood pressure ≥ 110mmHg during the study;

4. Serious violations of inclusion and exclusion criteria;

5. Poor compliance to the protocal;

6. Lost of follow-up;

7. Other reasonsFor subjects withdrew from the experiment, the researchers should ask whether there is occurrence of any adverse event, and request the completion of the last interview of the participants as far as possible. Laboratory tests should be conducted and adverse events are recorded, and adverse events should be tracked.

# Research design

## Research types and design principles

The study design is a randomized, double-blind, placebo-controlled, multi-center and phase I clinical trial.

After selection of the qualified 260 subjects, ther are randomly assigned to the following one of two groups at a ratio of 1:1:

The test group A: Allisartan Isoproxil tablet of 240 mg (130 cases)

The control group B: Allisartan Isoproxil placebo tablet of 240 mg (130 cases)

According to the clinical evaluation principles of anti-hypertensive drug E12A of the International Conference on Harmonization of technical requirements for registration of pharmaceuticals for human use (ICH), the middle and low risk patients with primary hypertension with ages of 18 ~ 70 years old are selected as the research population; Based on the effects of the different doses of the research drug on blood pressure that has been conducted in II stage and the exploration of the relationship between dose and effect, to confirm the absolute antihypertensive efficacy of the research drugs, the present study selects clinical objective only using fixed dose of Allisartan Isoproxil of 240 mg, and the parallel controlled study is conducted using the corresponding placebo; The treatment period is set as 8 weeks (in the placebo-controlled study, the maximum application time of the placebo is not more than 10 weeks) to observe the comparative efficacy and safety of the research drugs and placebo. At the same time, in order to observe the long term efficacy and safety of the research drugs, all subjects will participate safety observation period of 56 weeks voluntarily after they finish the double-blind treatment of 8 weeks, during which each subject will receive daily treatment of 240 mg Allisartan Isoproxil tablet once a day.

## Randomization and blinding

### Randomized method

The qualified subjects are selected. Drug number will be distributed randomly and the drugs corresponding to the drug number are given by the research doctor according to the grouping of each subject in order from small to large, which cannot be arbitrarily or intentionally selected. The number of the drug is maintained stable throughout the test process of the subject.

### Rules of breaking blinding of the randomized double-blind trial

**1）Blinding and preservation of blinding sheet**

Package of software was analyzed in computer by the biostatistician independent of the implementation of the data management and statistical analysis in the present study by using the SAS 9.13. The random encoding was produced by using the stochastic approach in the experiment group and the control group according to a ratio of 1:1. The length of the selected block and the initial seed parameters of the random number were sealed in the bottom of the blind area together as confidential data. The drugs were encoded according to thses random number under the surveillance of the personnel unrelated to the present test. The drugs were used by various clinical research centers according to the distributed number of the drugs and the grouping sequence of the cases. The blind end of two copies were sealed and stored in the sponsor’s department and the national drug clinical trial institution Office, the Third Xiangya Hospital of Central South University, the blind end should not be opened during the test.

**2）Enforcement of the blind method**

This study is a double-blind design. The extrinsic features of the research drug and placebo pills are exactly the same. The quantity of the research drug and the extrinsic features of the research drug taken by each subject are completely the same. In order to ensure that the batch number of the drugs used by each subject can be traced the source and ensure the blind test, the batch number of the research drug corresponding to each subject was recorded in the blind end when the research drugs were encoded blindly.

**3）Regulations of breaking blinding**

At the same time of encoding the research drugs, an emergency message was prepared for each drug number by the biostatisticians producing blinding end. The exact group of the number of the drug was sealed in the letter envelope. The emergency letters accompanied with the drugs of the corresponding numbers were distributed to various clinical research units by the researchers in charge of the unit. Emergency letter can only be used for emergency. They will be unsealed only the medication conditions should be understood and the treatment measures should be taken such as serious adverse events.

If the emergency envelope is opened, the exposing blind time, date and the reasons must be recorded on the envelope and the name of the people uncovering the blind should be signed off, and the Hangzhou Tigermed Co. Ltd. (CRO) company should be informed immediately. Once emergency letter is opened, the case is treated as the shedding case.

At the end of the study, all the emergency letters (opened or not) must be called back together with the completed case report form. The emergency letters should be checked when inspector visits each time to determine the envelope is not opened freely, or the number of envelops that have been opened is recorded clearly.

Blind can not be broken after double-blind treatment of 8 weeks. All participants will take part in a safety observation period of 56 weeks under blind states. The time of blind exposure is determined according to the requirements of the data statistics and analysis procedures of the double-blind treatments.

## Experimental steps and stages

The present study includes a 2-week cleaning period and a 8-week double-blind randomized treatment period. After treatments for 2 weeks, 4 weeks and 8 weeks, interviews are needed. There is a safety observation period with 56 weeks after finishing of the double-blind, randomized treatment period. One interview is needed to be done every 8 weeks.

The informed consents should be signed by the subjects before screening.

### Screening/cleaning periods (Interview 1, -3 weeks ~ 0 week)

Three weeks before the treatment with the research drug is given, and before the subjects take placebo (that is -3 weeks to -2 weeks), the following screening assessment projects should be completed:

- - Demographic data: including gender and ages (date of birth) of the subjects;
  - Height, weight, waist circumference, smoking and drinking status;
  - Collecting of the disease history: inquire and record the history of hypertension of the subjects (including the disease time of the blood pressure and levels of hypertension), family history, complications/diseases and their treatment histories, other previous disease and treatment history, allergic history;
  - Physical examination: physical examinations of all body systems are conducted;
  - Resting sitting blood pressure and heart rate measurements;
  - Laboratory and auxiliary examinations: including urine pregnancy test (limited to women of childbearing age), whole blood test, urine routine test test, liver function, renal function, fasting blood glucose, blood lipid, blood electrolyte and 12-lead electrocardiogram.

Preliminary screening of low and middle risk patients with primary hypertension, and basically meeting the inclusion criteria but not with the exclusion criteria, the patients were into the placebo washout period of 2 weeks (that is -2 weeks ~ 0 week) with distribution and taking 2 weeks of placebo and diary cards of the subjects at the same time.

In addition, recording the signed informed consent and the adverse events and concomitant treatment during placebo wash out period.

### Baseline period (Interview 2, 0 week)

After the subjects taking the placebo for 2 weeks, the following aspects should be conducted:

- - Rest sitting blood pressure and heart rate were measured: the measurement method is the same as that of the screening period;
  - Auditing the inclusion/exclusion criteria, the qualified subjects were distributed with random numbers according to the order of grouping from small to large order (i.e. drug number);
  - Distribution of drug of the first ~ the second week treatment period (including 3 days of alternate drug) according to the random numbers issued;
  - Recovery of the research drugs in the placebo washout period of 2 weeks;
  - Transfer and recording of diary cards of subjects;
  - Recording adverse events and concomitant treatments.

### Treatment period (Interview 3, 2 weeks ± 3 days)

After the subjects taking 2 weeks of the research drugs in the double-blind randomized treatment period, the following asepects are needed:

- - Resting sitting blood pressure and heart rate measurement: the measurement method is the same as that of the screening period;
  - Distribution of drug of the third ~ the fourth week treatment period (including 3 days of alternate drug) according to the random numbers issued;
  - Recovery of the research drugs in the placebo washout period of the first and the second week;
  - Transfer and recording of diary cards of subjects;
  - Recording adverse events and concomitant treatments.

### The treatment period (Interview 4, 4 weeks ± 3 days)

After the subjects taking 4 weeks the research drugs in the double-blind randomized treatment period, the following aspects should be done:

- - Resting sitting blood pressure and heart rate were measured: the measurement method is the same as that of the screening period;
  - Laboratory examinations: urine routine test test, liver function, renal function and serum electrolyte;
  - According to the distributed random numbers, the research drugs for the fifth ~ eighth week treatment period were given (including 6 days of alternative drug);
  - Recovery and counting of the third ~ fourth week remaining reserch drugs;
  - Transfer and recording of the diary cards of subjects;
  - Recording adverse events and concomitant treatments.

### Finishing of the random therapy (Interview 5, 8 weeks ± 3 days)

After the subjects taking 8 weeks of the research drugs in the double-blind randomized treatment period, the following aspects are needed:

- - Physical examinations: physical examination of all body systems including weight;
  - Resting sitting blood pressure and heart rate were measured: the measurement method was as the same as that of the screening period;
  - Laboratory and auxiliary examinations: including urine pregnancy test (limited to women of childbearing age), whole blood test, urine routine test test, liver function, renal function, fasting blood glucose, blood lipid, blood electrolyte and 12-lead electrocardiogram.
  - Recovery of the fifth ~ eighth week remaining study drug and counting;
  - Distribution of Allisartan Isoproxil tablets for 9-weeks doses;
  - Recovery, transfer and recording of the diary cards of subjects;
  - Recording adverse events and concomitant treatments.

### The safety observation period (Interview 6-11, 9-56 weeks, every interview for 8 weeks, the window period ± 7 days)

In order to observe the long-term efficacy and safety of the research drugs, all the subjects would participate in the safety observation period research of 56 weeks voluntarily after they finish the double-blind randomized study of 8 weeks, during which each subject will receive 240 mg Allisartan Isoproxil tablet treatment once a day, and the subjects should visit the doctor every 8 weeks:

- - Resting sitting blood pressure and heart rate were measured: the measurement method is the same as that of the screening period;
  - Laboratory examinations: urine routine test test, liver function, renal function and serum electrolyte (if the laboratory indicators are abnormal in the observation of former 8 weeks, please reexamine; if it is normal, please judge freely whether reexamination is necessary according to the circumstances);
  - Distribution of Allisartan Isoproxil tablets for 9 weeks doses;
  - Residual drug recovery of the past 8 weeks and counting;
  - Recovery, transfer and recording of the diary cards of the subjects;
  - Recording adverse events and concomitant treatments.

The interview 9, namely the interview of the subjects taking the research drugs for 32 weeks during the safety observation period, in addition to the above items, the following aspects are also needed to be conducted:

- - Physical examination: physical examination of all body systems including weight;
  - Laboratory examinations: urine routine test test, liver function, renal function and serum electrolyte;
  - Laboratory and auxiliary examinations including whole blood test, fasting blood glucose, blood lipid, blood electrolyte and a 12-lead electrocardiogram.

### The finishing of the safety observation period (Interview 12, the sixty-fourth week ± 7 days)

After the participants complete the observation period of 56 weeks of treatment, the following aspects are needed:

- - Physical examination: physical examination of all body systems including weight;
  - Resting sitting blood pressure and heart rate were measured: the measurement method is as the same as that of the screening period;
  - Laboratory and auxiliary examinations: including urine pregnancy test (limited to women of childbearing age), whole blood test, urine routine test test, liver function, kidney function, fasting blood glucose, blood lipid, blood electrolyte and a 12-lead electrocardiogram.
  - Recovery of the fifty-seventh ~ 64th week remaining study drug and counting;
  - Recovery and transfer and recording of diary cards of the subjects;
  - Recording adverse events and concomitant treatments.

### The unplanned outside interview

If subjects need extra interviews or early exiting test, the measures having been taken by researchers (including laboratory test results) should be recorded on the original case history and the unplanned interview table in the case report table.

In addition, when the subjects exit the tests early before the interview 5 or enter the safety observation period early to end the safety observation period, the following aspects should be done as far as possible:

- - Physical examination: physical examination of all body systems;
  - Resting sitting blood pressure and heart rate were measured: the measurement method is as the same as that of the screening period;
  - Laboratory and auxiliary examination: including urine pregnancy test (limited to women of childbearing age), whole blood test, urine routine test test, liver function, renal function, fasting blood glucose, blood lipid, blood electrolyte and a 12-lead electrocardiogram;
  - Recovery of all the remaining study drug and counting;
  - Recovery, transcfer and recording of the diary cards of the subjects (only applicable to the subjects during the double-blind study);
  - Recording adverse events and concomitant treatments.

# Research drugs

## Drug name, dosage form

Test drug: Allisartan Isoproxil tablet (ALS-3), specification of 80mg/tablet (each tablet containing 80mg ALS-3), produced and provided by Shenzhen Salubris Pharmaceuticals Co.,Ltd

and the Shanghai ALLIST medicine technology limited, batch number: 20090301.

Placebo: Allisartan Isoproxil placebo tablet, specifications 80mg/tablet, produced and provided by Shenzhen Salubris Pharmaceuticals Co.,Ltd and the Shanghai ALLIST medicine technology limited, batch number: 20090303.

## Medication methods

**The double-blind treatment period**

Test group: Allisartan Isoproxil tablet 240 mg, 1 time a day, oral administration of half an hour before breakfast fasting for 8 weeks.

Control group: Allisartan Isoproxil placebo tablet 240mg, 1 time a day, oral administration of half an hour before breakfast fasting for 8 weeks.

**Safety observation period**

Allisartan Isoproxil tablet 240 mg, 1 time a day, oral administration of half an hour before breakfast fasting for 56 weeks.

## Storage conditions

Below 30℃, dry places for preservation.

## Drug packaging and labeling

### Packaging of drugs in wash-out period

Two weeks cleaning period of research drug is 1 independent packaging. In order to ensure that the subjects are blinded, the packaging is not indicated the cleaning period of drugs and labeled as "Allisartan Isoproxil clinical trial drug (the first – the second week)". Only the screening number is filled but not the drug number. Drug packaging: Allisartan Isoproxil placebo 80mg/tablet; each plate contains 3 Allisartan Isoproxil placebo, and a total of 17 plates are included (including 3 days alternative drugs); fasting oral administration half an hour before breakfast, 1 time a day, each time for 1 plate.

### Packaging of drugs during the treatment period

The packaging of the research drugs include large packaging, big packaging, middle packaging and small packaging.

Each of the large package (big bog) includes the research drugs for 8 weeks randomized double-blind treatment period on a subject, which is labeled as "Allisartan Isoproxil clinical trial drug (the third – the tenth week), containing 4 large packages (a total of 68 plates).

Each of the large package (small bag) includes the research drugs for 2 weeks of the treatment period on a subject, which is labeled as "Allisartan Isoproxil clinical trial drug (the x – the x week), containing 3 middle packages (a total of 17 plates).

The middle packag (Aluminium plastic composite film bags) is divided into two kinds: one is "7 days of medication" (a total of 7 plates) and another is a "3 days of alternative medicine" (a total of 3 plates).

Each small packag is 1 aluminum plate. Each plate contains Allisartan Isoproxil tablet/placebo of 3 tablets, which is 1 day of medication.

### Labels of the research drugs

The labels of the research drugs include the labels of the research drugs in the cleaning period, the research drug of the randomised double-blind treatment period and the research drug of the safety observation period (Appendix 3).

## Drug distribution and checking

The cleaning period drugs for 2 weeks were distributed by the research physician in charge of Drug Administration to the preliminarily screened eligible subjects, which was recorded. After finishing the cleaning period for 2 weeks, the physicians were responsible for the recovery and counting the residual drugs and empty packaging inventory of the selected subjects.

After the cleaning period, the included subjects were further screened for the qualified subjects according to the research protocol. According to the selected sequence from small to large, random numbers (i.e. drug number) were distributed, and 1 ~ 2 weeks of the research drugs corresponding to the drug number in the randomized double-blind treatment period were distributed. The amount of the distributed drugs was recorded.

After finishing the treatment for 2 weeks, the subjects were interviewed by returning to the research center according to the research protocol. The research physician were responsible for the recovery and counting of the remaining drug number and empty package of all subjects, and 3-4 weeks randomized double-blind research drugs in the treatment period were distributed. The number of recovery and distributed drugs were recorded.

After finishing the treatment for 4 weeks, the subjects were interviewed by returning to the research center according to the research protocol. The research physician were responsible for the recovery and counting of the remaining drug number and empty package of all subjects, and the fifth to the eighth week randomized double-blind research drugs in the treatment period were distributed. The number of recovery and distributed drugs were recorded.

After finishing the treatment for 8 weeks, the subjects were interviewed by returning to the research center according to the research protocol. The research physician were responsible for the recovery and counting of the remaining drug number and empty package of all subjects, and the ninth to the sixteenth week randomized double-blind research drugs in the treatment period were distributed. The number of recovery and distributed drugs were recorded.

In the 56 weeks of safety observation period, the subjects were interviewed by returning to the research center according to the research protocol. The research physician were responsible for the recovery and counting of the remaining drug number and empty package of all subjects, and the ninth to the sixteenth week randomized double-blind research drugs in the treatment period were distributed. The number of recovery and distributed drugs were recorded.

After finishing the 56 weeks of safety observation period, the subjects were interviewed by returning to the research center according to the research protocol. The research physician were responsible for the recovery and counting of the remaining drug number and empty package of all subjects.

Whether the subjects complete the study or not, the untaken drugs and empty packaging must be returned to the researchers. Researchers should keep all the returned drugs and empty packaging for the recording until the CRO company together recovery of the not distributed products.

## The principle of dose selection and dose adjustment

**The selection principle of the doses of the research drugs and dosage adjustment:**

The reserch dose of the present clinical trial is set as 240mg, mainly taking the following factors into consideration:

In the already finished phase II clinical study of Allisartan Isoproxil, from the angle of the pressure amplitude and standard rate, the antihypertensive efficacy of the Allisartan Isoproxil tablets of 80mg, 160mg and 240mg was comparable to that of the 50mg losartan potassium, of which the antihypertensive effect of the Allisartan Isoproxil in the 240 mg group was similar to that fo the 50 mg losartan potassium, and the antihypertensive consistency in different treatment times was good, and the fluctuation was small. In security, there was no significant difference in the adverse events, adverse reactions and laboratory tests, vital signs and physical examinations between the groups of Allisartan Isoproxil with doses of 80mg, 160mg and 240mg and the group of losartan potassium with 50mg. However, of the 3 doses of Allisartan Isoproxil, the group of 240 mg Allisartan Isoproxil tended to show stronger roles in reducing blood lipid and renal protective effect. And in the indexes of liver function, there was no significant difference before and after treatment and between different groups. In the aspects of comprehensive efficacy and safety performance, this study chooses the 240mg dose as the test dose.

Throughout the study, the dose remained unchanged and there is no dose adjustment.

# Treatment of subjects

## Research drugs

All the preliminarily screened qualified patients intending to participate in the study, they all take the research drugs in the 2 weeks cleaning period :

Placebo: Allisartan Isoproxil placebo tablet: 80mg, 3 tablets.

After the 2 weeks cleaning period, 260 subjects meeting grouping conditions were randomly assigned to the following one of the two groups:

- Test group A: Allisartan Isoproxil tablet 240mg (Allisartan Isoproxil tablets 80mg, 3 tablets), once a day, fasting oral administration half an hour before breakfast;
- Placebo group B: Allisartan Isoproxil placebo tablet 240mg (Allisartan Isoproxil placebo tablets 80mg, 3 tablets), once a day, fasting oral administration half an hour before breakfast;

All the subjects finished the double-blind randomized studies after 8 weeks of treatment. All subjects participated in the safety observation research with a period of 56 weeks on a voluntary basis, during which, the participants took Allisartan Isoproxil tablets 240mg (Allisartan Isoproxil tablets 80mg, 3 tablets), once a day, fasting oral administration half an hour before breakfast.

## Combined application of drugs

Combined application of Losartan potassium with potassium supplement pills or isokalaemic diuretic (such as spironolactone, triamterene, amiloride etc.) may cause the increase in serum potassium level. Losartan potassium can increase the toxicity of lithium. Fluconazole can inhibit the transformation of losartan potassium into active metabolites of EXP3174. Similar to other anti hypertensive drugs, the non-steroidal anti-inflammatory drug indometacin can decrease the antihypertensive effects of losartan potassium. Rifampicin had obvious induction roles to the present drug and its metabolites EXP3174, reducing AUC and serum concentration of the drug and decreasing its efficacy. Ephedra contains ephedrine and pseudoephedrine, which can reduce the antihypertensive effects of the drugs. For the patients with hypertension treated with losartan potassium, formulations containing ephedra should be avoided.

EXP3174 is the active medicinal product of ALS-3. The active metabolites of losartan potassium playing main pharmacodynamic actions are also EXP3174. The matters needing attention in the specification of losartan potassium have very important reference value to ALS-3. Therefore, the above drugs refered to are not allowed to be used in the study period (including the cleaning period of 2 weeks), and any other drugs for treating hypertension are prohibited at the same time.

The interaction of drugs in the clinical application of Allisartan Isoproxil that may happen has not been studied.

All the concomitant medications were recorded in the corresponding position of the case report form.

## Subjects compliances

For the compliances of the medication orders, the drug use situation was calculated according to the recovery quantity of the distributed drugs combined with the diary cards of the subjects;

Actual dose of drug taken by subjects

Compliance＝

The dose of drug should be taken by the subjects

If the compliance of subject < 80％, or > 120％, indicating that the compliance is poor, not entering the per protocol set for analysis.

# Efficacy assessment

## Primary outcome

- The changes from baseline in trough mean seated DBP (23 to 26 hours after the morning dose) to the end of the 8-week double-blind treatment period.

Baseline refers to the sitting blood pressure before giving the research drug in the randomized double-blind treatment period and after 2 weeks cleaning period.

## Secondary outcome

- The change of trough mean seated DBP after 4 weeks treatment
- The change of trough mean seated SBP after 4 ,8weeks treatment
- Effective rate after 4,8 weeks treatment
- The change of trough mean seated SBP/DBP after 4, 8weeks treatment in effective patient.

Note: Effective respond was defined as the seated SBP/DBP<140/85mmHg, or seated SBP decrease≥20mmHg and/or seated DBP≥10mmHg.

# Safety assessment

## Safety index

### Physical examination

Comprehensive physical examinations are all conducted before treatment, after 8 weeks randomized double-blind treatment period and after the safety observation of 56 weeks.

### Laboratory and auxiliary examination

Before treatment, 8 week randomized double-blind treatment period and observed for 56 weeks after the end of the safety after the end of the period, were

The urine pregnancy test (limited to women of childbearing age), whole blood test, urine routine test test, blood biochemistry (including liver function, renal function, blood glucose, blood lipid and electrolyte) and 12-lead ECG examination were all conducted before treatment, after 8 weeks randomized double-blind treatment period and after the safety observation of 56 weeks.

Because there were renal toxicity of the research drugs in animal experiments, and the adverse reactions of liver were decreased by decreasing the metabolism of losartan reduces *in vivo*. In the present study, examinations of urine routine test test, liver function, renal function and blood electrolyte were conducted with the follow-up 8 weeks once in the fourth week of the randomized double-blind treatment period and the safety observation period to assess the safety of the research drugs.

## Adverse events

### Definition of adverse events

Adverse events (Adverse Event AE) refers to that patients or clinical trial subjects occur adverse medical events after receiving a drug, but it is not necessarily a causal relationship with this treatment.

Significant adverse event (Significant Adverse Event) refers to the addition of serious adverse events, the occurrence of any adverse events caused by application of the corresponding medical measures (such as withdrawal of drugs, reduced dose and symptomatic treatment) and hematologic or other laboratory examination abnormality.

### Observation, recording and reporting of adverse events/serious adverse events

Researchers should explain to subjects in detail, and ask subjects to reflect the changes of disease after treatment faithfully. Physicians should avoid suggestive question. During the test, it should pay close attention to the adverse events observed, cause analysis, judgment, and follow-up observation and recording.

For any adverse events during the trial, the occurring time, symptoms, severity, duration, treatment measures and prognosis should be recorded in the case report form and to evaluate its correlation with the experimental drugs, and the researchers do a detailed record, signing and dating.

### Criteria of severity degrees of adverse events/serious adverse events

All adverse events/significant adverse events should be evaluated severity by researchers, and rating should be done according to the criteria of 1 grade (mild), grade 2 (moderate), grade 3 (severe) and 4 grade (life-threatening) standards.

Grade 1 (mild): feeling unwell, but not interfering with daily activities, easily enduring.

Grade 2 (moderate): degree of discomfort is enough to reduce or affect daily activities, may require medical intervention.

Grade 3 (severe): unable to work or engage in daily activities, require medical intervention.

Grade 4 (life-threatening): a great possibility of death when occurrence of the events.

### Criteria of relationship between experimental drugs of adverse events/serious adverse events

Determination of the relationship between adverse events/signiciant adverse events and the test drugs:

Determination according to 5 grade criteria: that is certainly related, may be, may be independent, independent, be unable to be evaluted. The first two are adverse reactions, and the incidence rate of the adverse reaction is analyzed.

Certainly related: the appearance of reaction conforms to the reasonable time order after drug application, and the reaction conforms to the known reaction type of the suspected drugs; improvement after stopping of drug, occurrence of the reaction after repeated administration of the drug.

May be relatd: the appearance of reaction conforms to the reasonable time order after drug application, and the reaction conforms to the known reaction type of the suspected drugs; the patient's clinical status or other treatments may also induce this reaction.

Probably not related: the reaction is not in line with the reasonable time order after drug application, and the reaction is not in line with the known reaction type of the suspected; the patient's clinical status or other treatments may also induce this reaction.

Unrelated: the reaction does not comply with the time sequence after reasonable, a reaction type with non experimental drugs known reaction; the patient's clinical status or other treatments may cause the reaction, improve the state of disease or stop the elimination reaction to other treatments, the repeated use of other treatment response.

Unable to evaluate: the reaction has no relationship with the medication time, type and the drug known reaction similar to that of other drugs may also cause the same reaction.

### The treatment, follow-up and duration of cases of adverse events/serious adverse events

All adverse events/important adverse events, researchers should take the necessary measures according to their severity, and follow up to solve, returned to baseline level, proved unable to solve/permanent or death.

### The judgment and treatment of parameter abnormalities of laboratory test

Clinical significance of abnormal laboratory data of all researchers must obtain the trial period to evaluate. Must be complete and report any clinically significant laboratory abnormalities, and follow-up to the normal.

Clinical significance is defined as the researchers that have important clinical significance, require medical intervention or meet any abnormal data definition of serious adverse events ". If there are other clinical indications, clinical observation and evaluation, to determine the anomaly results significant or cause or monitoring of adverse events.

Abnormal laboratory can not explain the value, to repeat the examination, and follow-up to the value returned to normal reference range or reach the baseline, and / or find a reasonable explanation. If there is a clear explanation, please record in the case report form.

## Serious adverse events

### Definition of serious adverse event

Serious adverse events (Severe Adverse Event, SAE) refers to the following clinical events unforeseeable happen at any dose: death; life threatening; hospitalization or extend the time of hospitalization; lead to the loss of persistent or significant function, or lead to congenital malformations or birth defects.

### Recording and report procedure of serious adverse events

During the course of clinical trials, researchers must pay close attention to the occurrence of serious adverse events. Once the occurrence of serious adverse events, should immediately stop the test, and to take the necessary measures to ensure the safety of participants, at the same time in 24 hours by phone or fax report clinical trials unit ethics committee, State Food and Drug Administration (SFDA), sponsor and Hangzhou Tigermed Co. Ltd. (CRO). Serious adverse events following main contact person:

| SFDA | Registration Division  Fax.: 010-68313182  Safety supervision department  Fax.: 010-88363228 |
| --- | --- |
| The arbitrator: | Hangzhou Tigermed Co. Ltd.  Yajie Cao  Contact telephone number: 0731-4479357  Fax.: 0731-8850233 |

Researchers must fill out a serious adverse event report within 24 hours and fax to the State Food and drug administration. The relevant medical documents shall be recorded in the original file, including laboratory and auxiliary examination results report (such as: electrocardiogram etc.).

Researchers the serious adverse events occurred trying to analyze from a clinical perspective and identified with the test drug causality.

### Treatment, mode of follow-up and duration of the occurrence of serious adverse events

All serious adverse events, researchers should immediately take necessary and effective measures to solve, follow-up and returned to baseline level, proved unable to solve / permanent or death.

### Measures of subjects after pregnancy

During the study period subjects after pregnancy, must immediately stop clinical trials and told the researchers. In the treatment of study within 3 months after the end of pregnancy must also report to researchers. Researchers should put all the pregnant women with pregnancy report report to Hangzhou Tigermed Co. Ltd. in 24 hours (CRO) and sponsor. Researchers should provide the consultation, discussion on the risk of pregnancy and its influence on the fetus. Continued monitoring of patients to the end of pregnancy. The final results should be pregnancy providing research sponsor.

To participate in the study subjects were pregnant wife should also report to the researchers and the sponsor. To provide consultation and follow-up of the pregnant wife of.

# Data management

## The traceability of data, and fill in and hand over of case report form (CRF)

The most original record as the original medical records needs to save. Case report form data are derived from the original medical records, to be completed by the researchers, each selected get a random number of cases must be completed case report form. The case report form by the CRA examination, the first stock exchange statistics unit of data, data entry and management work. The first joint after the transfer case report table of contents will not be modified.

## Design and establishment of database

Establish database of clinical trials with trace management; record all the information in the CRF table. Database format will be as far as possible with the format of the CRF table corresponding to facilitate entry.

## Data encode

Not applicable.

## Input and modification of data

Data entry and management by the Shanghai Tigermed Co. Ltd. (CRO) statistics department is responsible for, will designate special personnel data management, developing data management plan. To ensure the accuracy of the numerical data, using double entry and proofreading; adverse events and drug in combination with single input and manual check.

The present case report table in question, the data administrator will fill in question and answer form (DRQ), and send inquiries to the researchers through clinical research associate, researchers should answer as soon as possible and return the data administrator data modification according to the researcher's answer, confirmation and entry, when necessary, can be re issued DRQ.

## Data verification

The verification of the data will be divided into manual verification and system verification. Manual verification is the data administrator errors found by consistency, check the data logic and other means to produce DRQ. The system checks or procedural examination (Computerized edit check) refers to the verification, the data in the CRF by the method of the computer program including range, logic, consistency, scheme violated and deviation. The DRQ will be handed over to the CRA, the CRA to researchers to determine again. The amendment requires researchers to sign and date.

## Locking and unblinding of database

Confirmation and the blind review database correctly, by the main researchers, the applicant, statistical analysis of common database lock. Do not allow the database principle changes after locking. Find the data after lock problem, can be confirmed by the analysis program in statistical correction, and keeping a written record.

The data will be kept locked, the blind end of head of the unit staff, the applicant and the statistics personnel joint first exposing blind, to determine the order of A, B in which the group encoding each of the subjects belonging to, and make statistical analysis according to the statistics personnel database plan requirements for statistical analysis, and then the two open label, determine the trial group and control group. The statistical analysis report writing statistical units, mainly studies the test write research reports.

# Statistics

## Sample size estimation

This study is the research on high efficiency, the efficiency test of the main therapeutic effect index. A: bilateral α = 0.05, P = 0.20, the standard deviation of the difference between SD=7.8mmHg and D detected two groups of antihypertensive effect of = 3mmHg, two cases of the distribution ratio of 1:1, the estimated minimum sample size was 108, taking into account factors such as the research plan off, 260 cases into two groups, the experiment group and the control group with 130 cases in each. The estimation results of PASS2008 software sample:

**Numeric Results for Superiority Test (H0: D <= |E|; H1: D > |E|)**

**Test Statistic: T-Test**

|  |  | **Equivalence** | **Actual** | **Significance** |  | **Standard** | **Standard** |
| --- | --- | --- | --- | --- | --- | --- | --- |
|  |  | **Margin** | **Difference** | **Level** |  | **Deviation1** | **Deviation2** |
| **Power** | **N1/N2** | **(E)** | **(D)** | **(Alpha)** | **Beta** | **(SD1)** | **(SD2)** |
| 0.80229 | 108/108 | 0.00 | 3.00 | 0.02500 | 0.19771 | 7.80 | 7.80 |

## Definition and selection of analytic set

The full analysis set and statistical analysis conducted in accordance with the scheme set set and data security. The whole process of statistical data will be strict quality control to ensure the authenticity of the data, assessment.

Full analysis set (Full analysis set, FAS): according to the intention to treat (ITT) principle, the random into groups, and the subjects more than once medication administration record and to evaluate the effectiveness of the enrolled in the full analysis set. In the choice of full analysis set analysis, the missing value estimation, can adopt the LOCF (LOCF) estimation method. Full analysis set is the main evaluation of the effectiveness of the study population.

The project sets (Per-Protocol set PPS): with the scheme set includes all completed treatment provisions scheme or no serious violations of test subjects. The exact definition of serious violations of the scheme will ultimately determine the data audit, generally include the following situations (but are not limited to these cases): Do not meet the criteria, interference treatment in selected, poor compliance, and visit beyond the time window. PP set is a secondary analysis of the crowd's effectiveness, but the results such as the full analysis set is inconsistent, to the detailed analysis of the inconsistent results.

Safety data set (Safety Set, SS): the definition of safety data set for at least the subjects received a drug treatment.

## Hypothesis testing

Superiority test primary efficacy index:

***H*_0_:μ_treatment_-μ_placebo_≤0**

***H*_1_:μ_treatment_-μ_placebo_＞0**

**α=0.05(two setting)**

**μ_treatment_-μ_placebo_**

**u= Se ＞1.96**

The alpha level to H0, lower or test group and placebo hypotensive effect difference of 95%CI is greater than 0, it is considered that the experimental group is better than the placebo.

All other statistical tests using bilateral test P value of less than or equal to 0.05 can be considered statistically significant.

## Statistical methods

### Aalysis method

Statistical analysis using SAS9.13 statistical analysis software for calculation. Quantitative description of the calculation of mean and standard deviation, median, minimum value, maximum value. Classification index described by number and percentage of cases.

Completion of the study will be carried out descriptive statistics on the demographics and baseline characteristics were compared between the two groups in a balanced analysis between groups.

Analysis of the main outcome measures: the main efficacy index will adopt non inferiority test. In addition, also calculate each subject after 8 weeks of treatment, DBP decreased and the seat value groups (mean standard deviation, median, minimum and maximum), groups were compared using analysis of variance center effect considered.

Analysis of secondary efficacy indicators: in principle does not consider the center effect, by using variance analysis, chi square test or nonparametric statistical method for comparison between groups.

Analysis of safety evaluation, safety evaluation included adverse events / ADR / important adverse events or serious adverse events, physical examination and laboratory examination and a 12 lead electrocardiogram. Cases of adverse events during the detailed description of test, summary comparison of incidence of adverse events between the two groups. Statistical description and index of laboratory examination and necessary changes were compared between the two groups. A detailed description of laboratory indexes was normal before treatment after treatment of abnormal.

### Interim analysis

No interim analysis in this study.

# Experimental management

## Declaration

This clinical trial will strictly abide by the scheme, GCP and SOP regulations and requirements for.

## Ethics part

Researchers have a responsibility to provide the clinical trial protocol, to the ethics committee and informed consent and provided to the subjects of information, independent approval documents to obtain the implementation of the clinical research.

Clinical research must be obtained before the start of the documents of approval of the ethics committee. Instruments of ratification of the ethics committee shall be made in written form to the researchers, and then by the researchers will be approved copies of documents provided to the sponsor. The documents of approval shall be accompanied by all involved in the ethics committee approval documents on the list of members and their responsibilities.

In the clinical research process, any relevant clinical research of safety issues, such as the clinical research plan or subjects informed consent to changes and clinical studies of serious adverse events, must report to the ethics committee in a timely manner. Clinical study of the end or early termination must also report to the ethics committee.

## The verification of original data

The directly recorded in the case report form data (i.e., no written or electronic records prior data) and account for the identification of the original data, according to the plan prior to the prescribed in the monitoring plan, clear. Otherwise, as the lack of original data.

Researchers must properly handle all in clinical research data, so as to ensure the participation in clinical research subjects of rights and privacy. Researchers must agree to the auditor / Inspector / inspectors on clinical research documents required for inspection and verification, to verify the original data accuracy and understand the research progress. If not on the original record verification, the researchers should agree to assist the auditor / Inspector / inspectors on the quality of the data for further confirmation.

## Quality control and assurance

All products and materials used in clinical research must be based on the quality control as the prerequisite. The sponsor, the sponsor authorized personnel or related medical management institutions have the right to carry out inspection on clinical research, its purpose is to guarantee the authenticity of clinical research data and clinical research program in compliance with the provisions of. Clinical study participants will be notified of the test process will have the relevant personnel to check, but the patient privacy and data will be subject to strict protection.

## Informed consent / data protection protocol

Researchers have a responsibility to each person subject to explain the purpose of the clinical trials, and the benefits and potential risks, and clinical trial subjects signed informed consent. Before any clinical trial related procedures, informed consent must be obtained from subjects. For those who for whatever reason, cannot sign the informed consent of the subjects, must by their parents, legal guardians or protect people signed the informed consent. By signing the informed consent, participants must agree to allow clinical study of Auditor / Inspector / health surveys to check has been about clinical study of the original data, in order to determine the reliability of clinical study data.

Signed informed consent and indicate the signing date of the book of the original must be made by the researchers kept by the subjects in clinical trials, in case report forms and related test records also need to record the informed consent.

## Modification of clinical program

Clinical programme changes to sponsors and investigators involved in. Plan revision shall be submitted to the examination and approval of the ethics committee. Before you can execute the revised content.

## Cases Report form

Researchers must ensure completely and accurately fill the case report form. Each case report table records the data information of only a clinical research subject. All the wrongly filled data or text may not be altered, but it must be drawn by a single row, and then re-enter the correct data or text on the side, and signed the name and date modified. The original case report form is stored in the sponsor, and the research centers keep a copy of it. Data entry must use the original case report form.

## Clinical Research monitor

Clinical research associate has the responsibility to be familiar with all the researchers including the co-researchers participated in the present experiment including the distributor of the research drug and research nurse. The arbitrator must monitor the work condition of the research center at regular intervals. The research centers should be monitored at regular intervals on the grouping of the first test subject, during the research progress and at the end of the study, respectively.

The arbitrator has the responsibility to verify with researchers together whether the clinical trials are in strict accordance with clinical research program, and solve any problems during test process with the reserachers. The arbitrator also has the responsibility to conduct control verification of the records of the clinical research and the original medical recordsso as to guarantee the authenticity of clinical data.

The arbitrator will explain the verified results of the original data according to the standard operation procedures in detail. All the informed consents of all the clinical research subjects in the test group must be checked.

## Privacy of subjects

Researchers must ensure the maintenance of the privacy of the clinical trial subjects. In all of the submitted bid documents, only the patient number of the test and initials be used to determine the identities of the clinical trial subjects, but the full names of the subjects cannot be indicated. Researchers must properly keep the name and address of the relevant clinical trial subject and the corresponding group table. These group tables must be strictly kept confidential by researchers but not be submitted to the sponsor.

## Publications

Before the publication of the whole research results of the multi-center clinical study, the data of each center cannot be separately published.

Before the research results are published or announced, the sponsor retains the power to review the first draft.

## Archiving of data

In accordance with the requirement of the relevant regulations, researchers should properly preserve the original records of the clinical research. Researchers should save the clinical test data five years after termination of clinical trial.

# Appendix

## Appendix 1: Standard of diagnosis and risk stratification of blood pressure (Guidelines for the prevention and treatment of hypertension in China 2005 Edition)

Hypertension is defined as the follows: in the case of without the use of antihypertensive drugs, SBP ≥ 140mmHg and / or DBP ≥ 90mmHg, according to the level of blood pressure, hypertension can be divided into 1, 2, 3 grades. Systolic blood pressure ≥140mmHg and diastolic blood pressure <90mmHg is singly isolated as Simple systolic hypertension. The patient has a history of high blood pressure, is currently using antihypertensive drugs, although blood pressure is less than 140/90mmHg, it also should be diagnosed as hypertension.

**Definition and classification of blood pressure levels**

| Category | Systolic blood pressure (mmHg) | Diastolic blood pressure (mmHg) |
| --- | --- | --- |
| Normal blood pressure | <120 | <80 |
| Higher than normal | 120~139 | 80~89 |
| Hypertension: | ≥140 | ≥90 |
| Grade 1 hypertension (mild) | 140~159 | 90~99 |
| Grade 2 hypertension (moderate) | 160~179 | 100~109 |
| Grade 3 hypertension (severe) | ≥180 | ≥110 |
| Simple systolic hypertension | ≥140 | <90 |

If the patient's systolic and diastolic blood pressure levels were different, with higher grade shall prevail. Simple systolic hypertension can also be in accordance with the systolic blood pressure level is divided into 1, 2, 3.**Risk stratification of hypertension:**

Hypertension treatment decisions for patients not only according to the blood pressure levels, according to the following aspects: Other risk factors; the target organ damage or diabetes; the coexistence of clinical conditions such as heart disease and cerebrovascular disease and kidney disease patients; the personal status and economic conditions. In order to facilitate the risk stratification, WHO/ISH Guidelines Committee according to the Framingham Heart Study subjects 10 years of cardiovascular death, non fatal stroke and non fatal myocardial infarction data calculated with several risk factors for cardiovascular events after the influence of absolute risk (see table below).

**The factors affecting the prognosis of hypertension**

| The risk factors of cardiovascular disease factors of cardiovascular disease | Target organ damage (TOD) | Diabetes mellitus | The clinical condition of the coexistence of (ACC) |
| --- | --- | --- | --- |
| ·Systolic and diastolic blood pressure levels (1~3)level of systolic and diastolic blood pressure (1~3) | ·Left ventricular hypertrophy | Fasting blood glucose≥7.0mmol/L | Cerebral vascular diseases |
| Male >55 years | ECG | （126mg/dL） | Ischemic stroke |
| ·Female>65 years | Ultrasonic Beckoning or X-ray diagram：LVMI | Postprandial blood glucose≥11.1mmol/L | Cerebral hemorrhage |
| Smoking |  | （200mg/dL） | Transient ischemic attack |
| Dyslipidemia | ·Thickening of arterial walls |  | ·Heart diseases |
| TC≥5.7mmol/L | Carotid artery ultrasoundIMT≥0.9mm |  | History of myocardial infarction |
| （220mg/dL） |  |  | Angina pectoris |
| or LDL-C>3.6mmol/L | Ultrasonography or atherosclerotic plaque |  | Coronary revascularization |
| （140mg/dL） | ·A mild elevation of serum creatinine |  | Congestive heart failure |
| or HDL-C<1.0mmol/L | Male 115~133μmol/L |  | ·Kidney disease |
| （40mg/dL） | （1.3~1.5mg/dL） |  | Diabetic nephropathy |
| ·Family history of premature cardiovascular disease | MaleFemale107~124μmol/L |  | Impaired renal function (serum creatinine) |
| The first-degree relatives, age of onset <50岁 | （1.2~1.4mg/dL） |  | Male >133μmol/L |
| Abdominal obesity or obesity | ·Microalbuminuria |  | (1.5mg/dL) |
| Abdominal obesity *WC ≥85cm | Urinary albumin 30~300mg/24h |  | Female >124μmol/L |
| Female≥80cm | Albumin / creatinine ratio: |  | (1.4mg/dL) |
| ObesityBMI≥28kg/m2 | Male ≥22mg/g |  | Proteinuria（>300mg/24h） |
| Lack of physical activity | （2.5mg/mmol） |  | ·eripheral vascular disease |
| High sensitivity C reactive proteinor≥3mg/L | Female ≥31mg/g |  | Retinopathy: bleeding or oozing, |
| or C reactive protein≥10mg/L | （3.5mg/mmol） |  | Papillary edema |

TC: total cholol; LDC-C: low density lipoprotein cholol; HDL-C: high density lipoprotein cholol; LVMI: left ventricular mass index; IMT: the carotid artery intima-media thickness; BMI: WC: body mass index; waist circumference. * for obesity in China Working Group criteria

**Table 3: according to risk stratification, quantitative estimation of prognosis**

|  | Blood pressure (mmHg) | | |
| --- | --- | --- | --- |
| Other risk factors And history | Grade 1 hypertension | Grade 2 hypertension | Grade 3 hypertension |
|  | SBP140~159 | SBP160~179 | SBP≥180 |
|  | or DBP90~99 | or DBP100~109 | or DBP≥110 |
| have no other risk factors | Low risk | Medium risk | High risk |
| 1~2 risk factors | Medium risk | Medium risk | Very high risk |
| 3 risk factors, |  |  |  |
| Target organ damage or diabete | High risk | High risk | Very high risk |
| Clinical case IV coexist | Very high risk | Very high risk | Very high risk |

According to the risk degree of the patients were divided into the following 4 groups:

4.3.1 The low risk group

Age <55 years old, male and female aged <65 years old, stage 1 hypertension, no other risk factors, and belongs to the low risk group. Typically, <15% risk of major cardiovascular events in patients with 10 years of follow-up.

4.3.2 In the risk group

Stage 2 hypertension or 1~2 grade and 1~2 risk factors, patients should be given medication, medication should be observed before the start time, the doctor needs to be very careful judgment. Typically, the danger this group of patients followed 10 years of major cardiovascular events about 15%~20%, if the patients belonged to stage 1 hypertension, with a risk factor for cardiovascular risk occurred 10 years about 15%.

4.3.3 High risk group

Blood pressure level is 1 or 2, with 3 or more risk factors, and suffering from diabetes or hypertension target organ damage or level 3 but no other risk factors of high risk group patients. Typically, their risk of major adverse cardiovascular events after 10 years of about 20%~30%.

4.3.4 Very high risk group

Stage 3 hypertension also have 1 or more risk factors or with diabetes or hypertension target organ damage, or 1~3 level and clinical related diseases. Typically, the next 10 years the highest risk of major adverse cardiovascular events, as ≥ 30%, should be promptly began to treat the most positive.

The Framingham Heart Study data available in the US and Europe, not suitable for our country. The scholars of our country study of cardiovascular disease risk factors in most provinces in China queue (CMCS) data, that the CMCS cohort 10 year risk of coronary heart disease and risk factor levels were significantly lower than the Framingham study. But China's current lack of research data about the system, so the guide temporarily still used the Framingham data, when the data to be updated after summing up.

## Appendix 2: measurement methods of blood pressure and heart rate at resting sitting position in clinic

Measurement of blood pressure and heart rate clinic resting seat by researchers at standard conditions according to the unified standard of measurement. Sitting blood pressure measurements of specific requirements are as follows:

（1）Blood pressure measuring scale is 2mmHg as the unit of the mercury column sphygmomanometer.

（2）The use of the cuff appropriate size should be at least 80% upper arm cuff air bag package. Most of the arm circumference 25-35cm, should use the long 35cm, wide 12-13cm specification balloon cuff; obese or arm circumference big should use of large size cuff. Detection of SiDBP and SiSBP. The sound disappears when the pressure is DBP (Korotkoff fifth).

（3）The measured at least quiet rest 5 minutes before the measurements within 30 minutes of no smoking or drinking coffee, emptying the bladder.

（4）The measured take seat, had better sit chair, exposed the right arm (dominant side or subjects of the upper arm, upper arm) and heart at the same level.

（5）The cuff tightly tied on the subjects of the upper arm, lower margin cuff should 2.5cm in the elbow. The stethoscope probe brachial artery in pulse.

（6）Pneumatic quick measurement, so that the air bag pressure to radial artery pulse disappeared after the increase of 30mmHg (4.0kPa), and then at a constant rate (2mmHg/ seconds) slow release. In the slow heart rate, discharge rate should be slower. For diastolic blood pressure readings, quickly deflated to zero.

（7）Hear Korotkoff sound carefully during the bleeding process observation Korotkoff sound in phase I (first voice) and V phase (vanish) vertical height of the mercury column convex. Systolic blood pressure readings taken Korotkoff sound in phase I, diastolic blood pressure readings from the V phase Korotkoff sound. Korotkoff sound does not disappear, by Korotkoff sound in IV phase (Bian Yin) as a diastolic blood pressure.

（8）The unit of blood pressure mmHg in clinical use (mmHg).

（9）Should be separated by 2 minutes repeated measurement, taking the average of 3 readings. If the three arbitrary two systolic blood pressure difference of 10mmHg (including 10mmHg) above and / or diastolic pressure is above 5mmHg, add two times the mean blood pressure, remove the minimum and maximum values after three times, mean the integer (four to five homes).

（10）Blood pressure measurements should be performed in standard condition, namely, every day in the same time, the ipsilateral arm, with the same blood pressure gauge, by the same person measurement. All blood pressure should be measured at 8:00 in the morning to 10:00, namely the medication for 24 hours, before taking a dose of the drug, to measure blood pressure trough levels. If subjects had received the study drug day dose, arrangement of measuring the valley level on the second day of blood pressure should be.

The seat of heart rate measuring specific requirements are as follows:

Measurement of heart rate during the second and third measurements of sitting blood pressure 2 minutes interval (1 minutes).

## Appendix 3: Label of research drugs

**The cleaning period of drugs**

小标签用于每板药物（1天药物）Small labels for each plate (1 days drugs) drugs

Clinical study for use Protocal No.：TG0912ALS-3

Aili candesartan cilexetil phase II clinical trial

Specifications: 3 sheets / plates 2010.12.24 Effective date: 2010.12.24

Usage and dosage: half an hour before breakfast fasting oral, once a day, every time 1 plate

Shenzhen Salubris Pharmaceuticals Co.,Ltd

Shanghai ALLIST Medicine Technology Co., Ltd.

Clinical study for use Protocal No.TG0912ALS-3 7 days of medication

Aili candesartan cilexetil phase II clinical trial

Specifications: 3 sheets / plates 2010.12.24 Effective date: 2010.12.24

Packaging: plastic composite film bags, 7 plate / bag

Usage and dosage: half an hour before breakfast fasting oral, once a day, every time 1 plate

Please put the child is not easy to contact

Shenzhen Salubris Pharmaceuticals Co.,Ltd

Shanghai ALLIST Medicine Technology Co., Ltd.

Label for each bag of drugs (drug for 7 days)

Clinical study for use Protocal No.：TG0912ALS-3 3 days preparing medication

Aili candesartan cilexetil phase II clinical trial

Specifications: 3 sheets / plates 2010.12.24 Effective date: 2010.12.24

Packaging: plastic composite film bags, 3 plate / bag

Usage and dosage: half an hour before breakfast fasting oral, once a day, every time 1 plate

Please put the child is not easy to contact

Shenzhen Salubris Pharmaceuticals Co.,Ltd

Shanghai ALLIST Medicine Technology Co., Ltd.

Label for each bag of drugs (3 days standby drug)

Clinical study for use Protocal No.：TG0912ALS-3

Aili candesartan cilexetil phase II clinical trial medication (1-2 weeks)

Screening of No.： Subjects initials:

Date of issue: year month day

Specifications: 3 sheets / plates Period of validity: 2010.12.24

The batch number:20090301、20090303

Packing: carton, 3 bag / box, a total of 17 plate

Usage and dosage: half an hour before breakfast fasting oral, once a day, every time 1 plate

Storage conditions: 30 ℃, dry, dark storage condition

Please put all the remaining drugs and packaging to researchers

Please put the child is not easy to contact

Shenzhen Salubris Pharmaceuticals Co.,Ltd

Shanghai ALLIST Medicine Technology Co., Ltd.

Big label for each box (2 weeks drugs) drugs

**Study drug treatment randomized double blind**

Clinical study for use Protocal No.：TG0912ALS-3

Aili candesartan cilexetil phase II clinical trial

Drug No.: Specifications: 3 sheets / plates Effective date: 2010.12.24

Usage and dosage: half an hour before breakfast fasting oral, once a day, every time 1 plate

Shenzhen Salubris Pharmaceuticals Co.,Ltd

Shanghai ALLIST Medicine Technology Co., Ltd.

Small labels for each plate (1 days drugs) drugs

Clinical study for use Protocal No.：TG0912ALS-3 7 days of medication

Aili candesartan cilexetil phase II clinical trial

Drug No. Specifications: 3 sheets / plates Period of validity:2010.12.24

Packaging: plastic composite film bags, 7 plate / bag

Usage and dosage: half an hour before breakfast fasting oral, once a day, every time 1 plate

Please put the child is not easy to contact

Shenzhen Salubris Pharmaceuticals Co.,Ltd

Shanghai ALLIST Medicine Technology Co., Ltd.

Label for each bag of drugs (drug for 7 days)

Clinical study for use Protocal No.：TG0912ALS-3 3 days preparing medication

Aili candesartan cilexetil phase II clinical trial

Drug No. ： Specifications: 3 sheets / plates Period of validity: 2010.12.24

Packaging: plastic composite film bags, 3 plate / bag

Usage and dosage: half an hour before breakfast fasting oral, once a day, every time 1 plate

Please put the child is not easy to contact

Shenzhen Salubris Pharmaceuticals Co.,Ltd

Shanghai ALLIST Medicine Technology Co., Ltd.

Label for each bag of drugs (drug for 3 days)

Clinical study for use Protocal No.：TG0912ALS-3

Aili candesartan cilexetil phase II clinical trial medication (first week)

Drug No.： Subjects initials:

Date of issue: year month day

Specifications: 3 sheets / plates Period of validity: 2010.12.24

20090301、20090303 Batch number: 20090301, 20090303

Packing: carton, 3 bag / box, a total of 17 plate

Usage and dosage: half an hour before breakfast fasting oral, once a day, every time 1 plate

Storage conditions: 30 ℃, dry, dark storage condition

Please put all the remaining drugs and packaging to researchers

Please put the child is not easy to contact

Shenzhen Salubris Pharmaceuticals Co.,Ltd

Shanghai ALLIST Medicine Technology Co., Ltd.

Label for each small box of drugs (drug for 2 weeks)

Clinical study for use TG0912ALS-3 Protocol number：TG0912ALS-3

Aili candesartan cilexetil phase II clinical trial (third - 10 weeks)

Number of drugs Initials of subjects

Specifications: 3 sheets / plates Period of validity: 2010.12.24

The batch number: 20090301、20090303

Package description: Big box, box 4 / box, 3 bag / box, a total of 68 plate

Usage and dosage: half an hour before breakfast fasting oral, once a day, every time 1 plate

Storage conditions: 30 ℃, dry, dark storage condition

Please put all the remaining drugs and packaging to researchers

Please put the child is not easy to contact.

Shenzhen Salubris Pharmaceuticals Co.,Ltd

Shanghai ALLIST Medicine Technology Co., Ltd.

Increase the label for each large box of drugs (8 week)

The security tag pending observation period of study drugs.

# Reference

1. Guidelines for the prevention and treatment of hypertension in China 2005 Edition.Beijing：People's Medical Publishing House,2006 (in Chinese)
2. ICH Steering Committee (2000). Principles for clinical evaluation of new antihypertensive drugs E12A. ICH Steering Committee, principle document.
3. Guidelines of antihypertensive drugs in clinical trials.（2rd Edition ），March,2007 (in Chinese)
4. The safety and effective of olmesartan compared with losartan for 8 weeks in Grade 1 and 2 primary hypertensive patients in China. Chinese Journal of Cardiology. 2006，34（10）：877-881(in Chinese)
5. Jun-ren Zhu, Shi-ying Cai, Weilong-Fan, et al. The summary of the safety and effective of olmesartan in Grade 1 and 2 primary hypertensive patients in China. Chinese journal of new drugs and clinical. 2006，25（3）：236-237(in Chinese)
6. Ji-zhen Guo, Yue-sheng Qian, Ai-qun Ma, et al. The safety and effective of Irbesartan in primary hypertensive patients in China. Chinese journal of new drugs and clinical. 2002，21（11）：645-648(in Chinese)
7. Jin Bo, Jun-ren Zhu, Shi-ying Cai, et al. The safety and effective of Telmisartan in primary hypertensive patients at low-medium risk. Chinese Journal of Cardiology. 2002，30（12）：738-742(in Chinese)
8. Ning-ling Sun, Hong-yi Wang, Shi-wen Wang, et al. The comparision of Irbesartan and valsartan in primary hypertensive patients at low-medium risk. Chin J Clin Pharmacol. 2001，11（2）：87-90(in Chinese)
9. Jie Huang, Ji-zhen Guo, Ping Tao, et al. The safety and effective of losartan in primary hypertensive patients at low-medium risk. Chinese Journal of Cardiology. 1999，27（3）：196-199(in Chinese)
10. William J. Elliott , David A. Calhoun , Paul T. DeLucca ,et al.Losartan Versus Valsartan in the Treatment of Patientswith Mild to Moderate Essential Hypertension: Data from a Multicenter, Randomized, Double-Blind, 12-Week Trial, Clinical Therapeutics,2001,23(8):1166-1179
11. JM Mallion, JP Siche, Y Lacourcière, and the Telmisartan Blood Pressure Monitoring Group,ABPM comparison of the antihypertensive profiles of the selective angiotensin II receptor antagonists telmisartan and losartan in patients with mild-to-moderate hypertension, Journal of Human Hypertension (1999) 13, 657–664

# Signature page of the protocol

| The main research subjects:  The Third Xiangya Hospital of Central South University  Professor Hong Yuan | | | | | |  |
| --- | --- | --- | --- | --- | --- | --- |
| Signature: |  | |  |  | |  |
| Date: |  | |  |  | |  |
|  |  | |  |  | |  |
|  |  | |  |  | |  |
|  |  | |  |  | |  |
|  |  | |  |  | |  |
| The sponsor:  Shenzhen Salubris Pharmaceuticals Co.,Ltd  Shanghai ALLIST Medicine Technology Co., Ltd.  The chief medical officer Zhigang Bi | | | | | |  |
| Signature: |  |  | | |  | |
| Date: |  |  | | |  | |
|  |  |  | | |  | |
|  |  |  | | |  | |
|  |  |  | | |  | |
| Statisticians: Shanghai Tigermed Co. Ltd.  Chief biostatistician Binghua Su | | | | | |  |
| Signature: |  | |  |  | |  |
| Date: |  | |  |  | |  |
|  |  | |  |  | |  |
|  |  | |  |  | |  |

**Declaration of Property for Allisartan Isoproxil**

Allisartan Isoproxil was jointly developed by Shenzhen Salubris Pharmaceuticals Co.,Ltd and Shanghai ALLIST Medicine Technology Co., Ltd. ,and now the related property of Allisartan Isoproxil belongs to Shenzhen Salubris Pharmaceuticals Co.,Ltd.
