# Supplementary material for: A Randomized, Double Blind, Placebo-Controlled, Multicenter Phase II Trial of Allisartan Isoproxil in Essential Hypertensive Population at Low-Medium Risk
Source: PLoS One. 2015 Feb 18;10(2):e0117560. doi: 10.1371/journal.pone.0117560 (PMC4333341; doi:10.1371/journal.pone.0117560)
Supplement: S1 Table — (DOC) [file pone.0117560.s003.doc]

**Table S1. Clinical Trial Sites, Principal Investigators, and IRB**

| **Principal Investigators** | **Study Site** | **Institutional Review Board (IRB)** ^*^ |
| --- | --- | --- |
| **Hong Yuan** | *Center of Clinical Pharmacology, the Third Xiangya hospital, Central South University, Changsha* | Institutional Review Board, The Third Xiangya Hospital of Central South University, 138 Tong-Zi-Po Road, Changsha, Hunan(410013) (No.0915) |
| **Guo-gang Zhang** | *Department of Cardiovascular Medicine, Xiangya Hospital, Central South University, Changsha* | Institutional Review Board of Xiangya Hospital，Central South University, Xiangya Road 87#, Changsha, Hunan(410008)(No.20090602) |
| **Shui-ping Zhao** | *Department of Cardiology, the Second Xiangya Hospital,Central South University, Changsha* | Institutional Review Board, The Third Xiangya Hospital of Central South University, 138 Tong-Zi-Po Road, Changsha, Hunan(410013) (No.0915) |
| **Ying Guo** | *Department of Cardiology, Hunan Provincial People's Hospital, Changsha* | Institutional Review Board, Hunan Provincial People’s Hospital, 61 Jie-Fang-Xi Road, Changsha, Hunan(410005) (No.2009-14) |
| **Shi-juan Lu** | *Department of Cardiology, Haikou City People's Hospital, Haikou* | Institutional Review Board, Haikou City People's Hospital, 43 Renmin Road, Haikou(570208)(No.2009-03) |
| **Jian-lin Ma** | *Department of Cardiology, Hainan Provincial People's Hospital, Haikou* | Institutional Review Board, Hainan Provincial People's Hospital, 19 Xiu-Hua Road,Haikou,Hainan(570311)(No.200906) |
| **Fan-bo Meng** | *Department of Cardiology, China Japan Union Hospital of Jilin University, Changchun* | Institutional Review Board, China Japan Union Hospital of Jilin University, 126 Xian-Tai Road, Changchun,(130033)(No.2009-06) |
| **Ping Chen** | *Department of Cardiology, Shantou Central Hospital, Shantou* | Institutional Review Board, Shantou Central Hospital, 114# waima Road, Shantou, (515031)(No.2009-03) |

* This study was conducted in accordance with the ethical principles of the Declaration of Helsinki
