# Supplementary material for: A Randomized, Double Blind, Placebo-Controlled, Multicenter Phase II Trial of Allisartan Isoproxil in Essential Hypertensive Population at Low-Medium Risk
Source: PLoS One. 2015 Feb 18;10(2):e0117560. doi: 10.1371/journal.pone.0117560 (PMC4333341; doi:10.1371/journal.pone.0117560)
Supplement: S2 Table — (DOC) [file pone.0117560.s004.doc]

**Table S2. Requirements for Entry into the Phase-II Allisartan Isoproxil Clinical Study**

| **A. Inclusion Criteria:** |
| --- |
| 1. Male and female aged 18 to 70 years old |
| 2. BMI within 18.5-26kg/m^2^ |
| 3. Mild to moderate essential hypertension according to the diagnostic criteria of China (2005) |
| 4. The mean seated blood pressure after 2-week washout period with placebo (the blood pressure should be detected 3 times every 2 minutes after 5-minute resting in sitting position and then take the mean value) should be 140mmHg ≤SBP<180mmHg and 90mmHg ≤DBP< 110mmHg |
| 5. Able and willing to provide signed informed consent |
| **B. Exclusion Criteria:** |
| 1. Having been diagnosed or suspected as secondary hypertension |
| 2. The difference of systolic blood pressure in seated place measured for three consecutive times is greater than 20 mmHg, and the difference of diastolic pressure is greater than 10 mmHg |
| 3. Sick sinus syndrome, II~III atrial ventricular block, atrial flutter, atrial fibrillation, or other malignant arrhythmia |
| 4. Aneurysm in conducting artery or dissecting aneurysm, percutaneous tranluminal coronary angioplasty (PTCA) or other cardiac surgery |
| 5.  Unstable angina pectoris, acute myocardial infarction, cardiac failure, and cerebral accidents within recent 6 months |
| 6. Asthma or moderate to severe chronic obstructive lung disease |
| 7. Uncontrolled diabetes, FBG≥11mmol/L or with complications (degenerative nephritis and peripheral neuropathy) |
| 8. Renal arterial stenosis |
| 9. Renal inadequacy ( Cr>1.5×ULN (the upper limit of the normal range) |
| 10. Serious hepatopathy or hepatic inadequacy (ALT, AST or TBIL> 2×ULN) |
| 11. Electrolyte disturbances (blood potassium or sodium abnormality) |
| 12. Subjects with lower blood volume |
| 13. Female subjects with pregnancy or in lactation |
| 14. Subjects with drug or alcohol dependence |
| 15. Concomitant treatment with other drugs which will affect the blood pressure |
| 16. Subjects with resistant hypertension |
| 17. Allergy to any ingredient in the study |
| 18.attend other clinical trail within 3 month |
| 19. Suspected as white overcoat hypertension according to the judgment of investigators |
| 20. Subjects are thought unsuitable for the study by investigators |
