# Supplementary material for: A Randomized, Double Blind, Placebo-Controlled, Multicenter Phase II Trial of Allisartan Isoproxil in Essential Hypertensive Population at Low-Medium Risk
Source: PLoS One. 2015 Feb 18;10(2):e0117560. doi: 10.1371/journal.pone.0117560 (PMC4333341; doi:10.1371/journal.pone.0117560)
Supplement: S3 Table — (DOC) [file pone.0117560.s005.doc]

**Table S3. List of protocol changes in the study**

| Change time | Revised parts | Content modification | Reasons |
| --- | --- | --- | --- |
| 20/05/2009 | Exclusion Criteria No 4 | Add ‘Left heart hypertrophy diagnosed by ECG’ | Avoid potential risk in specific person |
| 20/05/2009 | Exclusion Criteria No 5 | ‘past disease history’ change into ‘within recent 6 months for unstable angina pectoris, acute myocardial infarction, cardiac failure, and cerebral accidents’ | Make the definition more clear |
| 20/05/2009 | Exclusion Criteria No 8 | Add ‘peripheral vascular disease’ | Avoid potential risk in specific person |
| 20/05/2009 | Exclusion Criteria No 9 | Renal dysfunction define into ‘creatinine higher than normal level’ | Make the definition more clear |
| 20/05/2009 | Exclusion Criteria No 11 | Electrolyte disorder define into ‘especially abnormal of potassium and Sodium with clinical meaning’ | Make the definition more clear |
| 06/08/2009 | Clinical Trial Site | Add two Clinical Trial Sites: ‘Department of Cardiology, China Japan Union Hospital of Jilin University, Changchun’ and ‘Department of Cardiology, Shantou Central Hospital Shantou’ | To guarantee finishing the study on time |
| 06/08/2009 | Inclusion Criteria No 2 | BMI rage change into 18.5-26kg/m^2^ | Make the range more rigorous |
