# Supplementary material for: A Randomized, Double Blind, Placebo-Controlled, Multicenter Phase II Trial of Allisartan Isoproxil in Essential Hypertensive Population at Low-Medium Risk
Source: PLoS One. 2015 Feb 18;10(2):e0117560. doi: 10.1371/journal.pone.0117560 (PMC4333341; doi:10.1371/journal.pone.0117560)
Supplement: S4 Table — (DOC) [file pone.0117560.s006.doc]

Table S4. Most Common Clinical Adverse Experiences (incidence >3% in any one group)*

|  | Allisartan Isoproxil | Placebo | All patients |
| --- | --- | --- | --- |
| albuminuria | 6 (4.4%) | 4 (2.9%) | 10 (3.6%) |
| hypercholesteremia | 5 (3.6%) | 1 (0.7%) | 6 (2.2%) |
| leucocyturia | 5 (3.6%) | 1 (0.7%) | 6 (2.2%) |
| hypertriglyceridemia | 9 (6.6%) | 4 (2.9%) | 13 (4.7%) |
| dizziness | 4 (2.9%) | 7 (5.1%) | 11 (4.0%) |
| headache | 5 (3.6%) | 3 (2.2%) | 8 (2.9%) |
| increases in aminopherase | 7 (5.1%) | 10 (7.2%) | 17 (6.2%) |

* The frequency of each adverse events was comparable in Allisartan Isoproxil and placebo group (*P*＞0.05).
